# Supplementary material for: Disease-related income and economic productivity loss in New Zealand: A longitudinal analysis of linked individual-level data
Source: PLoS Med. 2021 Nov 30;18(11):e1003848. doi: 10.1371/journal.pmed.1003848 (PMC8631646; doi:10.1371/journal.pmed.1003848)
Supplement: S1 Table — (DOCX) [file pmed.1003848.s001.docx]

Supplementary Table 1: Disease and condition case definitions

| **Major Group** | **Detailed group** | **Sub-group** | **Source data** | **Case definition rules for first diagnostic date** | **Code type** | **Codes** | **Mortality**  **- Underlying cause of death** |
| --- | --- | --- | --- | --- | --- | --- | --- |
| **Infections** |  |  | NMDS | First hospitalisation with diagnosis for each tax year | ICD-10-AM | A00 – A09, A15 – A21, A23 – A28, A30 – A33, A35 – A44, A46, A48 – A49, A51 – A60, A63 – A66, A68 – A71, A74 – A75, A77 – A83, A85 - A95, A98 – A99, B00 – B02, B05 – B09, B15 – B27, B30, B33 - B56, B58 – B60, B64 – B69, B71, B73 – B83, B85 – B92, B94 – B97, B99, G00 – G07, G09, H00.0, H05.0, H10, H13, H60.0 – H60.1, H60.3, H65 – H67, H70, J00 – J06, J09 – J18, J20 – J22, J36, J39.0 – J39.1, J85 – J86, L00 – L04, L08, L30.3, M00 – M01, M46.20, M46.22 – M46.30, M46.32 – M46.39, M46.50 – M46.58, M60.0, M65.02 – M65.08, M65.1, M71.01 – M71.09, M71.11 – M71.19, M72.5, M72.6, M86, N10, N30.0, N30.8, N34, N39.0, N41.0 – N41.2, N43.1, N45, N48.2, N70 – N74, N77.0, N77.1 | ICD-10-AM codes used |
| **Cancer** | **Lung cancer** |  | NZCR | First registration date of cancer.  Cancer considered no long prevalent if > 5 years since diagnosis. | ICD-10-AM | C33 – C34 | ICD-10-AM codes used |
|  | **Colorectal cancer** |  | NZCR | First registration date of cancer.  Cancer considered no long prevalent if > 8 years since diagnosis. | ICD-10-AM | C18 - C20 | ICD-10-AM codes used |
|  | **Breast cancer** |  | NZCR | First registration date of cancer. Female only.  Cancer considered no long prevalent if > 20 years since diagnosis. | ICD-10-AM | C50 | ICD-10-AM codes used |
|  | **Prostate cancer** |  | NZCR | First registration date of cancer. Male only.  Cancer considered no long prevalent if > 20 years since diagnosis. | ICD-10-AM | C61 | ICD-10-AM codes used |
|  | **Other cancer** |  | NZCR | First registration date of cancer.  Cancer considered no long prevalent if > 10 years since diagnosis. | ICD-10-AM | C00 – C17, C21 – C26, C30 – C32, C37 – C41, C43 – C49, C51 – C58, C60, C62 – C86, C88, C90 – C97 | ICD-10-AM codes used |
| **Endocrine** | **Type 2 diabetes mellitus (T2DM)** |  | NMDS | First date from either of the following rules:  Rule 1   1. First date for diabetes identified in VDR 2. Not included if any record of a non-T2DM diabetes diagnosis (principal or additional) in the NMDS.   Rule 2  First hospitalisation for T2DM | T2DM exclusion ICD-10-AM | E10, E12 – E14, O24.0, O24.2 – O243.9 | ICD-10-AM T2DM codes used |
|  |  |  |  |  | T2DM exclusion ICD-9-CM-A | 250.01, 250.03, 250.11, 250.13, 250.21, 250.23, 250.31, 250.33, 250.41, 250.43, 350.51, 250.53, 250.61, 250.71, 250.73, 250.81, 250.83, 250.91, 250.93 |  |
|  |  |  | VDR |  | T2DM ICD-10-AM | E11, O241 – O241.9 |  |
|  |  |  |  |  | T2DM ICD-9-CM-A | 250.00, 250.02, 520.10, 250.10, 250.12, 250.20, 250.22, 250.30, 250.32, 250.40, 250.42, 250.50, 250.52, 250.60, 250.62, 250.70, 250.72, 250.80, 250.82, 250.90, 250.92, 362.02, 648.01, 648.03, 648.04 |  |
|  | **Other endocrine** |  | NMDS | First hospitalisation with diagnosis | ICD-10-AM | D35.2, E00 – E07, E10, E12 – E16, E20 – E27, E30 – E35, E40 – E46, E50 – E56, E58 – E61, E63 – E65, E67 – E68, E70 – E80, E83, E85, E88 – E89, O24.0, O24.2 – O243.9 | ICD-10-AM codes used |
|  |  |  |  |  | ICD-9-CM-A | 227.3, 240 – 242, 244 – 246,  251 – 255, 257.1, 257.9, 258 – 267, 268.0 – 268.1, 268.9, 269 – 272, 273.8, 273.9, 275, 277.1 – 277.2, 277.31, 277.39, 277.4 – 277.6, 277.8 - 277.9, 278.00, 278.1 – 278.4, 278.8, 278.90 – 278.95, 278.8, 279.00 – 279.05, 279.10 – 279.13, 648.00, 648.02 |  |
| **Vascular and blood disorders** | **Ischaemic heart disease** |  | NMDS | First date from one of the following rules:  Rule 1  First hospitalisation with diagnosis  Rule 2  First hospitalisation with procedure  Rule 3  First dispensing date if an additional dispensing occurs on a different date within a year | ICD-10-AM | I20 – I25, I46 | ICD-10-AM codes used |
|  |  |  |  |  | ICD-9-CM-A | 410 – 414, 427.1, 427.41 – 427.42, 427.5,, 429.1, 429.2, 429.71, 429.79, 429.99 |  |
|  |  |  |  |  | ACHI | 3530400, 3530500, 3531000, 3531001, 3531002, 3849700, 3849701, 3849702, 3849703, 3849704, 3849705, 3849706, 3849707, 3850000, 3850001, 3850002, 3850003, 3850004, 3850300, 3850301, 3850302, 3850303, 3850304, 3863700, 9020100, 9020101, 9020102, 9020103 |  |
|  |  |  | PHARM |  | Chemical ID | - 1272 [Nicorandil] - 1577 [Glyceryl trinitrate]   - Excluding formulation of 26 (Oint 0.2%) - 1949 [Perhexiline maleate] - 2377 [Isosorbide dinitrate] - 2836 [Isosorbide mononitrate] |  |
|  | **Stroke (ischaemic & haemorrhagic)** |  | NMDS | First hospitalisation with diagnosis | ICD-10-AM | G54 – G46, I60 – I69 | ICD-10-AM codes used |
|  |  |  |  |  | ICD-9-CM-A | 430 - 438 |  |
|  | **Other cardiovascular disease** |  | NMDS | First hospitalisation with diagnosis | ICD-10-AM | I00 – I02, I05 – I11, I13, I15, I26 – I28, I30 – I45, I47 – I52, I70 – I74, I77 – I84, I86 – I89, I95, I97 – I99 | ICD-10-AM codes used |
|  |  |  |  |  | ICD-9-CM-A | 289.1 – 289.3, 390 – 398, 401 – 402, 404.01, 404.02, 404.11, 404.13, 404.91, 404.93, 405, 415 – 417, 420 – 426, 427.0, 427.2, 427.31 – 427.32, 427.60 – 427.61, 427.69, 427.81, 427.89, 427.9, 428, 429.0, 429.3 – 429.56, 429.81, 429.82, 429.89,440 – 444, 447 – 448, 451 – 454, 456 – 459, 997.1, 997.2 |  |
|  | **Blood disorders** |  | NMDS | First hospitalisation with diagnosis | ICD-10-AM | D45 – D46, D50 – D53, D55 – D64, D66 – D76, D80 – D84, D89 | ICD-10-AM codes used |
|  |  |  |  |  | ICD-9-CM-A | 238.4, 238.7, 273.0, 729.06, 279.09, 279.19, 279.2 – 279.4, 279.8 – 279.9, 280 – 285, 286.0 – 286.5, 286.7, 286.9, 287 – 288, 289.0, 289.4, 289.50, 289.51, 289.59, 289.6 – 289.7, 289.9 |  |
| **Mental Disorders** | **Anxiety and depressive disorders** |  | PRIMD | First hospitalisation or service contact with diagnosis | DSM IV | 296.2, 296.3, 300.0, 3oo.2 – 300.4, 308, 309.24, 309.28, 309.3, 309.4, 309.8, 309.9, 311 | Not included as cause of death |
|  |  |  | NMDS |  | ICD-10-AM | F32 – F33, F34.1, F40 – F43, F53.0 |  |
|  |  |  |  |  | ICD-9-CM-A | 296.2, 296.3, 296.82, 300.0, 300.10 – 300.14, 300.16, 300.2 – 300.8, 308, 309.0 – 309.1, 309.24, 309.28, 309.29, 309.3, 309.4, 309.8, 309.9, 311 |  |
|  | **Alcohol use disorders** |  | PRIMD | First hospitalisation or service contact with diagnosis | DSM IV | 291, 303, 305.0 |  |
|  |  |  | NMDS |  | ICD-10-AM | F10, X45 |  |
|  |  |  |  |  | ICD-9-CM-A | 291, 303, 305.0 |  |
|  | **Schizophrenia** |  | PRIMD | First hospitalisation or service contact with diagnosis | DSM IV | 295, 297 – 298, 301.22 |  |
|  |  |  | NMDS |  | ICD-10-AM | F20 – F25, F28 – F29, F53.1 |  |
|  |  |  |  |  | ICD-9-CM-A | 295, 297 – 298, 301.22 |  |
|  | **Other mental disorders** |  | PRIMD | First hospitalisation or service contact with diagnosis | DSM IV | 292, 293.9, 296.0, 296.4 – 296.9, 299, 300.1, 300.6 – 301.1, 301.20, 301.4 – 301.9, 302, 304, 305.1 – 305.7, 305.9, 306, 307.0 – 307.3, 307.44 – 307.47, 307.5 – 307.9, 309.21, 310, 312 – 315, V61.9 |  |
|  |  |  | NMDS |  | ICD-10-AM | F07 – F09, F11 – F19, F30 – F31, F34.0, F34.8 – F34.9, F38 – F39, F44 – F45, F48, F50, F51.1 – F51.5, F51.8 – F51.9, F52, F53.8 – F53.9, F55, F59, F60 – F66, F68 – F69, F80 – F84, F88- F95, F98 – F99 |  |
|  |  |  |  |  | ICD-9-CM-A | 292, 296.0, 296.1, 296.4 – 296.7, 296.80, 296.81, 296.89, 296.9, 299, 300.15, 300.19, 300.9 – 301.9, 302, 304, 305.1 – 305.9, 306, 307.0 – 307.3, 307.40, 307.43 – 307.47, 307.49, 307.5 – 307.7, 307.80, 307.89, 309.21 - 309.23, 310, 312 – 315, 648.44 |  |
| **Neurological conditions** | **Dementia** |  | NMDS | First date from either of the following rules:  Rule 1  First hospitalisation or service contact with diagnosis  Rule 2  First dispensing date | ICD-10-AM | F00 – F03, G30 – G31 | ICD-10-AM codes |
|  |  |  |  |  | ICD-9-CM-A | 290, 294.1, 294.9, 330, 331.0 – 331.3, 331.7 – 331.9 |  |
|  |  |  | PRIMD |  | DSM IV | 290, 294.1, 294.8 |  |
|  |  |  | PHARM |  | Chemical ID | 3750 [Rivastigmine]  3923 [Donepezil hydrochloride] |  |
|  | **Migraine** |  | NMDS | First date from either of the following rules:  Rule 1  First hospitalisation or service contact with diagnosis  Rule 2  First dispensing date | CD10 | G43 | Not included as cause of death |
|  |  |  |  |  | ICD-9-CM-A | 346 |  |
|  |  |  | PHARM |  | Chemical ID | 1214 [Dihydroergotamine mesylate]  1458 [Ergotamine tartrate]  1459 [Ergotamine tartrate with cyclizine]  1460 [Ergotamine tartrate with diphenhydramine]  1462 [Ergotamine tartrate with caffeine]  1815 [Metoclopramide hydrochloride with paracetamol]  2000 [Pizotifen]  2800 [Sumatriptan]  3876 [Rizatriptan] |  |
|  | **Primary insomnia** |  | NMDS | First hospitalisation or service contact with diagnosis | CD10 | F51.0, G47.0 | Not included as cause of death |
|  |  |  |  |  | ICD-9-CM-A | 701.41, 207.42 |  |
|  |  |  | PRIMD |  | DSM IV | 307.42, 780.52 |  |
|  | **Other neurological conditions** | **Earliest date of the following used for ‘other neurological conditions’:** | | | | | |
|  |  | **Epilepsy** | NMDS | First date from either of the following rules:  Rule 1   - First diagnosis date   Rule 2   - First dispensing date of group 1 chemicals   Rule 3   - First dispensing date of group 2 chemicals if there is also a diagnosis | ICD-10-AM | G40 – G41 | ICD-10-AM codes used |
|  |  |  |  |  | ICD-9-CM-A | 345 |  |
|  |  |  |  |  | Group 1 Chemical ID | 1004 [Vigabatrin], 1481 [Ethosuximide], 1956 [Phenobarbitone sodium], 1978 [Phenytoin sodium], 2041 [Primidone], 3354 [Phenobarbitone] |  |
|  |  |  | PHARM |  | Group 2 Chemical ID | 1002 [Lamotrigine], 1062 [Gabapentin], 1133 [Topiramate], 1217 [Carbamazepine], 1308 [Clobazam], 1316 [Clonazepam], 1397 [Diazepam], 2059 [Paraldehyde], 2166 [Sodium valproate] |  |
|  |  | **Intellectual impairment** | NMDS | First hospitalisation or service contact with diagnosis or involvement of specialised team | CD10 | F70 – F79 | Not included as cause of death |
|  |  |  |  |  | ICD-9-CM-A | 317 – 319 |  |
|  |  |  |  |  | NMDS Health Specialty Code | D60 – D74 |  |
|  |  |  | PRIMD |  | DSM IV | 317 - 319 |  |
|  |  |  |  |  | PRIMD Team Type | 12 |  |
|  |  | **Other** | NMDS | First hospitalisation or service contact with diagnosis | ICD-10-AM | F04 – F06, F70 – F73, F78 – F79, G08, G10 – GG14, G20 – G26, G32, G35 – G37, G40 – G41, G44, G47.1 – G47.2, G47.4, G47.8, G47.9,G50 – G64, G70 – G73, G81 – G83, G90 – G99, H81.4 | ICD-10-AM codes used |
|  |  |  |  |  | ICD-9-CM-A | 294.0, 294.8, 307.8, 325, 331.4, 332 – 337, 340 – 342, 344, 347 – 359, 780.50, 780.54, 780.55, 784.0, 997.09 |  |
|  |  |  | PRIMD |  | DSM IV | 293.0, 293.8, 294.0, 294.9, 332, 333, 347, 780.0, 780.54, 780.59 |  |
| **Sense organ disorders** | | | NMDS | First hospitalisation with diagnosis | ICD-10-AM | H00.1, H01 – H04, H05.1 – H05.9, H06, H11, H15 – H22, H25 – H28, H30 – H36, H40, H42 – H59, H60.2 -, H60.4 – H60.5, H60.8 – H60.9, H61 – H62, H68 – H69, H71 – H75, H80, H81.0 – H81.3, H81.8 – H81.9, H83, H90 – H95 | Not included as cause of death |
|  |  |  |  |  | ICD-9-CM-A | 053.2, 053.7, 054.4, 094.8, 360 – 361, 362.01, 362.1 – 362.9, 363 – 371, 372.4 – 372.9, 373.0, 373.12, 373.13, 373.2 – 373.6, 373.8 – 373.9, 374 – 375, 376.1 – 376.6, 376.8 – 376.9, 377 – 379, 380.00, 380.13 – 380.16, 380.2 – 380.5, 380.8 – 380.9, 381.5 – 381.9, 383.30 – 383.33, 384 - 389, 997.99 |  |
| **Respiratory disorders** | **Chronic obstructive pulmonary disease (COPD)** | | NMDS | First hospitalisation with diagnosis | ICD-10-AM | J40 – J44 | ICD-10-AM codes used |
|  |  |  |  |  | ICD-9-CM-A | 490 – 492, 495 - 496 |  |
|  | **Asthma** | | NMDS | First hospitalisation with diagnosis | ICD-10-AM | J45 – J46 | ICD-10-AM codes used |
|  |  |  |  |  | ICD-9-CM-A | 293 |  |
|  | **Other** | | NMDS | First hospitalisation with diagnosis | ICD-10-AM | D86, E66.2, E84, G47.30 – G47.33, G47.39, J30 – J35, J37 – J38, J39.2 – J39.3, J39.8 – K39.9, J47, J60 – J70, J80 – J82, J84, J90 – J96, J98 – J99 | ICD-10-AM codes used |
|  |  |  |  |  | ICD-9-CM-A | 135, 277.0, 470 – 474, 476 – 477, 478.0 – 478.1,  478.20, 478.25, 478.26, 478.29, 478.3 – 478.9, 494, 500 – 509, 511 – 512, 516 – 519, 780.51, 997.3 |  |
| **Gastrointestinal disorders (GI)** | **Upper GI disorder** | | NMDS | First hospitalisation with diagnosis | ICD-10-AM | K20 – K21, K22.1, K25 – K30 | ICD-10-AM codes used |
|  |  |  |  |  | ICD-9-CM-A | 530.10, 530.11, 530.19, 530.2, 530.81, 531 – 535, 536.8 |  |
|  | **Chronic Liver disease** | | NMDS | First hospitalisation with diagnosis | ICD-10-AM | K70, K71.0 –K71.1, K71.3 – K71.9, K72 – K76 | ICD-10-AM codes used |
|  |  |  |  |  | ICD-9-CM-A | 570 - 573 |  |
|  | **Other GI disorder** | | NMDS | First hospitalisation with diagnosis | ICD-10-AM | I85, K22.0, K22.2 – K22.9, K23, K31, K35 – K38, K40- K46, K50 – K52, K55 – K67, K71.2, K77, K80 – K83, K85 – K87, K90 – K93, T78.0 | ICD-10-AM codes used except N40 |
|  |  |  |  |  | ICD-9-CM-A | 040.2, 455, 530.0, 530.3 – 530.7, 530.82, 530.83, 530.9, 536.0 – 536.3, 536.9, 537, 540 – 543, 550 – 553, 555 – 558, 560, 562, 564 – 569, 574 – 579, 995.6, 997.4 |  |
| **Genitourinary (GU) disorders** | **Chronic kidney disease** | | NMDS | First hospitalisation with diagnosis | ICD-10-AM | I12, I13.1, N03 – N07, N11 - N13, N18 – N19, Q60 – Q61 | ICD-10-AM codes used |
|  |  |  |  |  | ICD-9-CM-A | 403, 404, 581 – 586, 590.00 – 590.01, 590.80, 591, 593.4, 593.6, 593.70 – 593.73, 599.6, 753.0 – 753.1 |  |
|  | **Other GU disorders** | | NMDS | First hospitalisation or service contact with diagnosis | ICD-10-AM | E29, N00 – N02, N08, N14 – N17, N20 – N23, N25 – N29, N30.1 – N30.4, N30.9, N31 – N33, N35 – N37, N39.1 – N39.9, N40 – N44, N46 – N51, N99 | ICD-10-AM codes used |
|  |  |  |  |  | ICD-9-CM-A | 257.0, 257.2, 257.8, 580, 581.81, 583.4, 583.81, 587 – 590, 592, 593.0 – 593.3, 593.5, 593.81 – 593.83, 593.89, 593.9, 594, 595.1 – 595.4, 595.82, 595.9, 596, 597.0, 597.80 -597.81, 598.0, 598.1, 598.8 – 598.9, 599.1 – 599.5, 599.7 – 599.9, 600, 601.3 – 601.4, 601.8 - 601.9, 602, 603.0, 603.8 - 603.9,604.91, 605 – 606, 607.0 – 607.1, 607.3, 607.8 – 607.9, 608, 625.6, 788.0, 788.31 |  |
|  |  |  | PRIMD |  | DSM IV | 607.84, 608.99 |  |
| **Reproductive disorders** | | | NMDS | Female only.  First hospitalisation or service contact with diagnosis | ICD-10-AM | D25, E28, N60 – N64, N75 – N76, N77.8, N80 – N98, N99.1 – N99.4, N99.8 – N99.9 | Not included as cause of death |
|  |  |  |  |  | ICD-9-CM-A | 218, 256, 298.2, 610-611, 616.10, 616.2 – 616.4, 616.50, 616.8, 616.9,617 – 624, 625.0 – 625.5, 625.8, 625.9, 626 – 629, 997.5 |  |
|  |  |  | PRIMD |  | DSM IV | 625 |  |
| **Skin disorders** | | | NMDS | First hospitalisation with diagnosis for each tax year | ICD-10-AM | L05, L10 – L13, L20 – L29, L30.0 – L30.2, L30.3 – L30.5, L30.8 – L30.9, L40 – L45, L50 – L60, L63 – L68, L70 – L75, L80 – L95, L97 – L99 | Not included as cause of death |
|  |  |  |  |  | ICD-9-CM-A | 136.0, 690.1, 691 – 693, 694.0 – 694.5, 694.60, 694.8 – 694.9, 695.0 – 695.4, 695.89, 695.9, 696.1, 696.2, 696.8, 697, 698, 701 – 709, 909.2 |  |
| **Musculoskeletal (MSK) disorders** | **Spinal disorders** | | NMDS | First hospitalisation with diagnosis | ICD-10-AM | M47, M48.0 – M48.2, M48.8 – M48.9, M50 – M51, M53 – M54 | Not included as cause of death |
|  |  |  |  |  | ICD-9-CM-A | 721.0 – 721.6, 721.8 – 722.7, 723.0 – 723.4, 723.6, 723.8, 723.9, 724, 729.2s |  |
|  | **Osteoarthritis** | | NMDS | First hospitalisation with diagnosis | ICD-10-AM | M15 – M19 | Not included as cause of death |
|  |  |  |  |  | ICD-9-CM-A | 715 |  |
|  | **Chronic MSK pain syndromes** | | NMDS | First hospitalisation with diagnosis | ICD-10-AM | M70.8 – M70.9, M75 – M77, M79.7 | Not included as cause of death |
|  |  |  |  |  | ICD-9-CM-A | 726.0 – 726.2, 726.31.726.32, 726.4, 726.5, 726.60 – 726.64, 726.69, 726.7 – 726.8, 726.90 |  |
|  | **Rheumatoid arthritis** | | NMDS | First hospitalisation with diagnosis | ICD-10-AM | M05 – M06 | Not included as cause of death |
|  |  |  |  |  | ICD-9-CM-A | 714.0 – 714.2, 714.33, 714.8, 714.9 |  |
|  | **Other MSK** | **First date used for Other MSK** | | | | | |
|  |  | **Gout** | NMDS | First date from one of the following rules:  Rule 1  First hospitalisation with diagnosis  Rule 2  First dispensing date of group 1 chemical  Rule 3  First dispensing date of group 2 chemical if there is no diagnosis of ‘ICD-10-AM exclusion’ in either NMDS or NCR within 24 months of dispensing | ICD-10-AM | M10 - 11 | Not included as cause of death |
|  |  |  |  |  | ICD-9-CM-A | 274, 712 |  |
|  |  |  | PHARM |  | Group 1 chemical ID | 1341 [Colchicine] |  |
|  |  |  |  |  | Group 2 chemical ID | 1026 [Allopurinol] |  |
|  |  |  | NZCR |  | ICD-10-AM exclusion | C81 – C96 |  |
|  |  | **Other** | NMDS | First hospitalisation with diagnosis | ICD-10-AM | M02, M07 – M08, M12 – M14, M20 – M25, M30 – M36, M40 – M43, M45 M46.0, M46.1, M46.4, M46.8, M46.9, M48.3, M48.4, M48.5, M49, M60.1, M60.2, M60.8, M60.9, M61 – M63, M65.2 – M65.4, M65.8 – M65.9, M66 – M68, M70.0 – M70.7, M71.2 – M71.5, M71.8 – M71.9, M72.40 – M72.49, M72.8 – M72.9, M79.0 – M79.6, M79.8 – M79.9, M80 – M85, M87 – M96, M99 | ICD-10-AM codes used |
|  |  |  |  |  |  | 099.3, 136.1, 268.2, 446, 696.0, 696.3 – 696.5, 710, 711.20, 711.23, 711.26, 711.30, 711.31, 711.34 – 711.36, 711.38, 711.3, 713, 714.30 – 714.32, 714.4, 716 – 718, 719.0 – 719.5, 719.8 – 719.9, 720, 721.7, 722.8, 723.5, 723.7, 725, 726.30, 726.33, 726.39, 726.65, 726.91, 727, 728.1 - 728.9, 729.0, 729.1, 729.3, 729.5 - 729.6, 729.8, 729.9, 731 – 739, 905.5, 996.78 |  |
| **Injury (nature of injury perspective)** | **Traumatic Brain Injury** |  | NMDS | First hospitalisation with diagnosis | ICD-10-AM | S06.03 – S06.05, S06.1 – S06.9, T90.5 | Death identified where the underlying cause was an injury. As there can be multiple nature of injuries then order of prioritisation from NZBD used to assign cause of death to the injury group. |
|  |  |  |  |  | ICD-9-CM-A | 800.03 – 800.05, 800.1 - 800.4, 800.53 – 800.55, 800.6 – 800.9, 801.1 – 801.4, 801.53 – 801.55, 801.6 – 801.9, 803.1 – 803.4, 803.34, 803.35, 803.5 - 803.9, 804.03 – 804.05, 804.1 – 804.4, 804.53 – 804.55, 804.6 – 804.9, 850.2 – 850.4, 851 – 853, 854.03 – 854.05, 854.13 – 854.15, 907.0 |  |
|  | **Internal injury** |  | NMDS | First hospitalisation with diagnosis for each tax year | ICD-10-AM | S25 – S27, S35 – S37, S39.6, T06.3, T06.5, T91.4, T91.5 |  |
|  | **Poison** |  | NMDS | First hospitalisation with diagnosis for each tax year | ICD-10-AM | T36 – T65, T96, T97 |  |
|  | **Other injury** |  | NMDS | First hospitalisation with diagnosis for each tax year | ICD-10-AM | S00 – S05, S06.00 – S06.02, S07 – S17, S19 – S24, S28.0, S29 – S34, S38, S39.0, S39.7 – S39.9, S40- S99, T00 – T05, T06.0 – T06.2, T06.4, T06.8, T07 – T31, T33 – T35, T66 – T71, T33 – T75, T78.1 – T78.9, T79 – T89, T90.0 – T90.4, T90.8 – T91.3, T91.8 – T91.9, T92 – T95, T98 |  |
