# Supplementary material for: Disease-related income and economic productivity loss in New Zealand: A longitudinal analysis of linked individual-level data
Source: PLoS Med. 2021 Nov 30;18(11):e1003848. doi: 10.1371/journal.pmed.1003848 (PMC8631646; doi:10.1371/journal.pmed.1003848)
Supplement: S2 Table — (DOCX) [file pmed.1003848.s002.docx]

Supplementary Table 2: Observation person-years and total income (2020 US$, millions) by diseases by phase

|  | **Person-years** | | | | **Income** | | | |
| --- | --- | --- | --- | --- | --- | --- | --- | --- |
|  | **Diagnosis** | **Prevalent** | **Death** | **Total** | **Diagnosis** | **Prevalent** | **Death** | **Total** |
| **Cancer** | **71,346** | **383,037** | **23,004** | **477,387** | **$1,690.65** | **$9,169.00** | **$140.64** | **$11,000.29** |
| Lung | 3,546 | 4,032 | 4,446 | 12,024 | $50.26 | $50.32 | $23.01 | $123.59 |
| Colorectal | 7,026 | 25,515 | 2,598 | 35,139 | $163.99 | $591.85 | $18.67 | $774.52 |
| Breast | 17,226 | 120,627 | 2,994 | 140,847 | $353.94 | $2,496.94 | $13.37 | $2,864.25 |
| Prostate | 10,062 | 42,180 | 468 | 52,710 | $318.30 | $1,303.99 | $2.92 | $1,625.21 |
| Other cancer | 35,769 | 196,896 | 12,507 | 245,172 | $852.86 | $4,847.92 | $82.67 | $5,783.45 |
| *Naïve sum* | *73,629* | *389,250* | *23,013* | 485,892 | $1,739.35 | $9,291.03 | $140.64 | $11,171.02 |
| *(%) > Overall cancer group* | *3.2%* | *1.6%* | *0.0%* |  | 2.9% | 1.3% | 0.0% |  |
| **Cardiovascular (CVD) and blood disorders** | **134,295** | **2,025,291** | **11,991** | **2,171,577** | **$3,268.61** | **$43,649.20** | **$98.52** | **$47,016.34** |
| Ischaemic heart disease | 47,853 | 423,162 | 6,813 | 477,828 | $1,067.59 | $8,890.66 | $63.56 | $10,021.81 |
| Stroke | 19,671 | 155,922 | 2,067 | 177,660 | $382.56 | $2,566.28 | $16.01 | $2,964.85 |
| Other CVD | 77,658 | 1,346,652 | 3,003 | 1,427,313 | $1,964.68 | $29,775.93 | $18.36 | $31,758.97 |
| Blood disorders | 19,614 | 518,478 | 105 | 538,197 | $351.18 | $9,047.98 | $0.60 | $9,399.76 |
| *Naïve sum* | *164,796* | *2,444,214* | *11,988* | 2,620,998 | $3,766.02 | $50,280.85 | $98.52 | $54,145.39 |
| *% > Overall CVD and blood group* | *22.7%* | *20.7%* | *0.0%* |  | 15.2% | 15.2% | 0.0% |  |
| **Mental illness** | **74,754** | **1,525,491** |  | **1,600,245** | **$1,057.55** | **$21,541.96** |  | **$22,599.52** |
| Anxiety and depressive disorders | 44,784 | 811,806 | ‡ | 856,590 | $623.39 | $11,655.56 | ‡ | $12,278.95 |
| Alcohol use disorders | 20,940 | 458,583 | ‡ | 479,523 | $305.37 | $6,679.34 | ‡ | $6,984.72 |
| Schizophrenia | 9,030 | 223,803 | ‡ | 232,833 | $57.20 | $1,311.34 | ‡ | $1,368.55 |
| Other mental illness | 27,945 | 656,166 | ‡ | 684,111 | $299.76 | $7,245.75 | ‡ | $7,545.51 |
| *Naïve sum* | *102,699* | *2,150,358* |  | 2,253,057 | $1,285.73 | $26,892.00 |  | $28,177.72 |
| *% > Overall mental illness group* | *37.4%* | *41.0%* |  |  | 21.6% | 24.8% |  |  |
| **Musculoskeletal (MSK) disorders** | **266,007** | **2,945,736** | **228** | **3,211,971** | **$7,218.76** | **$76,512.64** | **$1.03** | **$83,732.44** |
| Spinal disorders | 42,555 | 531,093 | ‡ | 573,648 | $923.94 | $11,022.11 | ‡ | $11,946.05 |
| Osteoarthritis | 38,607 | 231,216 | ‡ | 269,823 | $862.29 | $5,124.07 | ‡ | $5,986.37 |
| Chronic MSK pain syndrome | 23,970 | 220,707 | ‡ | 244,677 | $591.54 | $5,471.43 | ‡ | $6,062.98 |
| Rheumatoid arthritis | 1,404 | 27,912 | ‡ | 29,316 | $24.83 | $458.98 | ‡ | $483.81 |
| Other MSK disorders | 209,928 | 2,328,768 | 228 | 2,538,924 | $5,895.46 | $62,450.35 | $1.03 | $68,346.84 |
| *Naïve sum* | *316,464* | *3,339,696* | *228* | 3,656,388 | $8,298.07 | $84,526.94 | $1.03 | $92,826.04 |
| *% > Overall MSK disorders group* | *19.0%* | *13.4%* | *0.0%* |  | 15.0% | 10.5% | 0.0% |  |
| **Injury** | **355,956** | **79,608** | **8,127** | **443,691** | **$7,330.49** | **$1,374.17** | **$76.87** | **$8,781.53** |
| Traumatic brain injury | 3,666 | 79,713 | 2,085 | 85,464 | $62.43 | $1,374.78 | $20.58 | $1,457.79 |
| Internal injury | 5,037 | † | 690 | 5,727 | $109.51 | † | $9.05 | $118.55 |
| Poisoning | 34,266 | † | 1,716 | 35,982 | $415.18 | † | $11.71 | $426.89 |
| Other injury | 321,726 | † | 3,642 | 325,368 | $6,830.02 | † | $35.53 | $6,865.55 |
| *Naïve sum* | *364,695* | *79,713* | *8,133* | 452,541 | $7,417.14 | $1,374.78 | $76.87 | $8,868.79 |
| *% > Overall injury group* | *2.5%* | *0.1%* | *0.0%* |  | 1.2% | 0.0% | 0.0% |  |
| **Neurological Conditions** | **170,397** | **1,805,931** | **1,545** | **1,977,873** | **$4,097.31** | **$38,225.75** | **$3.19** | **$42,326.25** |
| Dementia | 1,968 | 13,323 | 231 | 15,522 | $10.12 | $55.46 | $0.09 | $65.68 |
| Migraine | 131,325 | 964,851 | ‡ | 1,096,176 | $3,332.09 | $23,524.06 | ‡ | $26,856.15 |
| Primary insomnia | 279 | 6,051 | ‡ | 6,330 | $3.56 | $73.96 | ‡ | $77.52 |
| Other neurological conditions | 50,184 | 922,881 | 1,317 | 974,382 | $987.19 | $16,165.27 | $3.09 | $17,155.55 |
| *Naïve sum* | *183,756* | *1,907,106* | *1,548* | 2,092,410 | $4,332.97 | $39,818.75 | $3.19 | $44,154.90 |
| *% > Overall neurological conditions group* | *7.8%* | *5.6%* | *0.2%* |  | 5.8% | 4.2% | 0.0% |  |
| **Respiratory disorders** | **69,468** | **1,669,395** | **2,226** | **1,741,089** | **$1,805.10** | **$39,136.14** | **$5.08** | **$40,946.33** |
| Chronic obstructive pulmonary disease | 10,419 | 111,714 | 1,470 | 123,603 | $122.87 | $1,444.93 | $2.03 | $1,569.84 |
| Asthma | 8,265 | 512,772 | 225 | 521,262 | $148.83 | $10,301.36 | $0.78 | $10,450.97 |
| Other respiratory disorders | 58,740 | 1,215,996 | 528 | 1,275,264 | $1,640.92 | $30,215.24 | $2.27 | $31,858.43 |
| *Naïve sum* | *77,424* | *1,840,482* | *2,223* | 1,920,129 | $1,912.62 | $41,961.54 | $5.08 | $43,879.24 |
| *% > Overall respiratory conditions group* | *11.5%* | *10.2%* | *-0.1%* |  | 6.0% | 7.2% | 0.0% |  |
| **Diabetes and other endocrine disorders** | **125,853** | **1,371,669** | **2,118** | **1,499,640** | **$2,747.70** | **$28,114.63** | **$7.39** | **$30,869.72** |
| Type 2 diabetes mellitus | 120,384 | 965,943 | 1,476 | 1,087,803 | $2,598.80 | $19,581.38 | $3.48 | $22,183.66 |
| Other endocrine disorders | 18,360 | 601,824 | 639 | 620,823 | $385.62 | $11,843.07 | $3.92 | $12,232.61 |
| *Naïve sum* | *138,744* | *1,567,767* | *2,115* | 1,708,626 | $2,984.43 | $31,424.46 | $7.39 | $34,416.28 |
| *% > Overall diabetes and other endocrine group* | *10.2%* | *14.3%* | *-0.1%* |  | 8.6% | 11.8% | 0.0% |  |
| **Reproductive disorders** | **109,071** | **2,168,235** | ‡ | **2,277,306** | **$2,556.44** | **$44,341.72** | ‡ | **$46,898.16** |
| **Gastrointestinal disorders** | **216,912** | **3,183,009** | **1,389** | **3,401,310** | **$5,745.80** | **$76,938.60** | **$5.54** | **$82,689.94** |
| Upper GI disorder | 40,434 | 592,458 | 126 | 633,018 | $855.05 | $12,111.83 | $0.54 | $12,967.42 |
| Chronic liver disease | 5,295 | 98,811 | 717 | 104,823 | $93.32 | $1,868.13 | $2.79 | $1,964.25 |
| Other GI disorders | 194,073 | 2,833,260 | 540 | 3,027,873 | $5,235.33 | $69,294.41 | $2.20 | $74,531.94 |
| *Naïve sum* | *239,802* | *3,524,529* | *1,383* | 3,765,714 | $6,183.70 | $83,274.37 | $5.54 | $89,463.61 |
| *% > Overall GI group* | *10.6%* | *10.7%* | *-0.4%* |  | 7.6% | 8.2% | 0.0% |  |
| **Infections** | **239,370** | **†** | **1,098** | **240,468** | **$4,397.25** |  | **$4.40** | **$4,401.65** |
| **Genitourinary (GU) disorders** | **66,138** | **955,653** | **384** | **1,022,175** | **$1,717.85** | **$22,676.90** | **$0.52** | **$24,395.27** |
| Chronic kidney disease | 20,769 | 274,578 | 315 | 295,662 | $462.32 | $5,328.01 | $0.37 | $5,790.71 |
| Other GU disorders | 53,478 | 774,549 | 69 | 828,096 | $1,436.40 | $19,096.02 | $0.15 | $20,532.57 |
| *Naïve sum* | *74,247* | *1,049,127* | *384* | 1,123,758 | $1,898.72 | $24,424.04 | $0.52 | $26,323.28 |
| *% > Overall GU group* | *12.3%* | *9.8%* | *0.0%* |  | 10.5% | 7.7% | 0.0% |  |
| **Skin disorders** | **45,750** | **†** | **‡** | **45,750** | **$1,099.08** | **†** | **‡** | **$1,099.08** |
| **Sensory disorders** | **54,042** | **694,491** | **‡** | **748,533** | **$1,253.43** | **$14,942.50** | **‡** | **$16,195.93** |

Diagnosis = first year of diagnosis; Prevalent = prevalent years of disease; Death yr = last year of life and dying of this disease.

Disease groups are not mutually exclusive, i.e. a single person-year observation with diagnoses of CVD, DM and Musculoskeletal will contribute observations (and expenditure) to: the three separate diseases, in one (only) of diagnosis, prevalent and last year of life phases.

† Acute events, not considered to have prevalent years.

‡ Death either not possible or too rare to present estimates.
