# Supplementary material for: Disease-related income and economic productivity loss in New Zealand: A longitudinal analysis of linked individual-level data
Source: PLoS Med. 2021 Nov 30;18(11):e1003848. doi: 10.1371/journal.pmed.1003848 (PMC8631646; doi:10.1371/journal.pmed.1003848)
Supplement: S3 Table — (DOCX) [file pmed.1003848.s003.docx]

Supplementary Table 3: Annual income loss (US$ 2020) for 14 and 40 diseases and conditions models predicted by fixed effects regression for 50-54 year olds (95% confidence intervals in parentheses)†

| **Diseases and conditions** | **Female** | | | | **Male** | | | |
| --- | --- | --- | --- | --- | --- | --- | --- | --- |
|  | **14 Diseases** | | **39 Diseases** | | **13 Diseases** | | **38 Diseases** | |
| Intercept (i.e. income of referent person with no disease) | $27,851 | ($27,771, $27,931) | $27,877 | ($27,797, $27,958) | $39,615 | ($39,509, $39,721) | $39,726 | ($39,620, $39,833) |
| **Year of diagnosis** | | | | | | | | |
| **Cancer** | -$1,594 | (-$1,806, -$1,381) |  |  | -$1,654 | (-$2,014, -$1,293) |  |  |
| Lung |  |  | -$1,983 | (-$2,737, -$1,229) |  |  | -$2,991 | (-$4,512, -$1,471) |
| Colorectal |  |  | -$2,085 | (-$2,783, -$1,388) |  |  | -$2,611 | (-$3,600, -$1,621) |
| Breast |  |  | -$1,766 | (-$2,101, -$1,432) |  |  |  |  |
| Prostate |  |  |  |  |  |  | -$1,027 | (-$1,652, -$403) |
| Other cancer |  |  | -$1,370 | (-$1,682, -$1,058) |  |  | -$1,565 | (-$2,076, -$1,053) |
| **Cardiovascular and blood disorders** |  |  |  |  | -$1,375 | (-$1,604, -$1,147) |  |  |
| IHD |  |  | -$1,525 | (-$1,811, -$1,239) |  |  | -$1,424 | (-$1,756, -$1,091) |
| Stroke |  |  | -$2,290 | (-$2,725, -$1,855) |  |  | -$2,392 | (-$2,970, -$1,814) |
| Other CVD |  |  | -$741 | (-$972, -$510) |  |  | -$949 | (-$1,266, -$633) |
| Blood disorders |  |  | -$1,307 | (-$1,711, -$903) |  |  | -$2,726 | (-$3,552, -$1,901) |
| **Mental illness** | -$4,160 | (-$4,459, -$3,861) |  |  | -$5,365 | (-$5,788, -$4,942) |  |  |
| Anxiety and depressive disorders |  |  | -$3,664 | (-$4,018, -$3,310) |  |  | -$5,018 | (-$5,605, -$4,431) |
| Alcohol use disorders |  |  | -$3,480 | (-$4,227, -$2,733) |  |  | -$4,796 | (-$5,493, -$4,099) |
| Schizophrenia |  |  | -$2,815 | (-$3,430, -$2,201) |  |  | -$3,651 | (-$4,587, -$2,714) |
| Other mental illness |  |  | -$2,881 | (-$3,333, -$2,429) |  |  | -$3,744 | (-$4,410, -$3,078) |
| **Musculoskeletal disorders** | -$1,340 | (-$1,480, -$1,200) |  |  | -$1,784 | (-$1,970, -$1,598) |  |  |
| Spinal disorders |  |  | -$1,737 | (-$2,061, -$1,412) |  |  | -$3,243 | (-$3,706, -$2,780) |
| Osteoarthritis |  |  | -$2,061 | (-$2,322, -$1,799) |  |  | -$2,801 | (-$3,201, -$2,401) |
| Chronic musculoskeletal pain syndrome |  |  | -$2,707 | (-$3,111, -$2,303) |  |  | -$3,894 | (-$4,407, -$3,380) |
| Rheumatoid arthritis |  |  | -$2,378 | (-$3,561, -$1,194) |  |  | -$777 | (-$3,703, $2,148) |
| Other MSK disorders |  |  | -$903 | (-$1,071, -$735) |  |  | -$1,154 | (-$1,363, -$944) |
| **Injury** | -$1,342 | (-$1,464, -$1,220) |  |  | -$1,840 | (-$1,994, -$1,686) |  |  |
| Traumatic brain injury |  |  | -$3,639 | (-$5,056, -$2,222) |  |  | -$2,859 | (-$4,257, -$1,461) |
| Internal injury |  |  | -$1,156 | (-$2,344, $31) |  |  | -$479 | (-$1,642, $684) |
| Poisoning |  |  | -$1,124 | (-$1,485, -$762) |  |  | -$1,341 | (-$1,869, -$813) |
| Other injury |  |  | -$1,251 | (-$1,379, -$1,122) |  |  | -$1,772 | (-$1,932, -$1,613) |
| **Neurological conditions** | -$190 | (-$347, -$33) |  |  | -$1,520 | (-$1,849, -$1,192) |  |  |
| Dementia |  |  | -$7,103 | (-$8,707, -$5,499) |  |  | -$8,882 | (-$11,056, -$6,709) |
| Migraine |  |  | $446 | ($265, $627) |  |  | -$147 | (-$595, $301) |
| Primary insomnia |  |  | -$243 | (-$3,904, $3,418) |  |  | -$2,470 | (-$6,344, $1,405) |
| Other neurological conditions |  |  | -$1,483 | (-$1,740, -$1,227) |  |  | -$2,593 | (-$3,045, -$2,142) |
| **Respiratory disorders** | -$297 | (-$558, -$35) |  |  | -$262 | (-$653, $130) |  |  |
| Chronic obstructive pulmonary disease |  |  | -$1,058 | (-$1,535, -$580) |  |  | -$1,749 | (-$2,535, -$963) |
| Asthma |  |  | -$624 | (-$1,279, $30) |  |  | -$652 | (-$2,116, $813) |
| Other respiratory disorders |  |  | -$81 | (-$384, $222) |  |  | -$77 | (-$507, $352) |
| **Diabetes and other endocrine disorders** | -$1,214 | (-$1,401, -$1,027) |  |  | -$901 | (-$1,179, -$622) |  |  |
| Type 2 diabetes mellitus |  |  | -$1,319 | (-$1,508, -$1,130) |  |  | -$865 | (-$1,142, -$589) |
| Other endocrine disorders |  |  | -$559 | (-$1,002, -$116) |  |  | -$1,044 | (-$1,817, -$270) |
| **Reproductive disorders** | -$186 | (-$347, -$26) | -$202 | (-$363, -$42) |  |  |  |  |
| **Gastrointestinal disorders** | -$539 | (-$688, -$391) |  |  | -$463 | (-$678, -$249) |  |  |
| Upper GI disorder |  |  | -$316 | (-$616, -$16) |  |  | -$203 | (-$662, $256) |
| Chronic liver disease |  |  | -$2,912 | (-$3,787, -$2,037) |  |  | -$3,015 | (-$4,206, -$1,824) |
| Other GI disorders |  |  | -$511 | (-$670, -$352) |  |  | -$509 | (-$735, -$283) |
| **Infections** | -$566 | (-$698, -$434) | -$505 | (-$637, -$374) | -$752 | (-$943, -$562) | -$655 | (-$845, -$465) |
| **Genitourinary disorders** | -$263 | (-$554, $29) |  |  | -$460 | (-$801, -$120) |  |  |
| Chronic kidney disease |  |  | -$1,063 | (-$1,573, -$554) |  |  | -$673 | (-$1,337, -$8) |
| Other GU disorders |  |  | -$10 | (-$354, $334) |  |  | -$419 | (-$787, -$52) |
| **Skin disorders** | -$151 | (-$411, $109) | -$147 | (-$406, $113) | -$209 | (-$664, $246) | -$195 | (-$650, $259) |
| **Sensory disorders** | -$1,072 | (-$1,333, -$812) | -$1,026 | (-$1,286, -$766) | -$1,160 | (-$1,559, -$760) | -$1,124 | (-$1,523, -$725) |
| **Last year of life if died from disease** | | | | | | | | |
| **Cancer** | -$13,110 | (-$13,678, -$12,543) |  |  | -$16,800 | (-$17,615, -$15,984) |  |  |
| Lung |  |  | -$12,170 | (-$13,408, -$10,932) |  |  | -$15,191 | (-$16,839, -$13,543) |
| Colorectal |  |  | -$14,192 | (-$16,026, -$12,357) |  |  | -$17,786 | (-$20,018, -$15,555) |
| Breast |  |  | -$13,349 | (-$14,489, -$12,210) |  |  |  |  |
| Prostate |  |  |  |  |  |  | -$15,529 | (-$19,448, -$11,609) |
| Other cancer |  |  | -$12,850 | (-$13,697, -$12,002) |  |  | -$16,571 | (-$17,630, -$15,512) |
| **Cardiovascular and blood disorders** | -$7,366 | (-$8,210, -$6,521) |  |  | -$12,037 | (-$12,816, -$11,258) |  |  |
| IHD |  |  | -$6,711 | (-$8,003, -$5,418) |  |  | -$11,698 | (-$12,653, -$10,743) |
| Stroke |  |  | -$8,278 | (-$9,881, -$6,675) |  |  | -$12,592 | (-$15,023, -$10,162) |
| Other CVD |  |  | -$5,522 | (-$7,045, -$3,999) |  |  | -$10,148 | (-$11,767, -$8,528) |
| Blood disorders |  |  | -$7,935 | (-$13,741, -$2,129) |  |  | -$15,944 | (-$23,272, -$8,615) |
| **Musculoskeletal disorders** | -$7,141 | (-$10,110, -$4,171) |  |  | -$9,582 | (-$18,324, -$840) |  |  |
| Other MSK disorders |  |  | -$6,667 | (-$9,611, -$3,724) |  |  | -$8,308 | (-$17,120, $504) |
| **Injury** | -$6,957 | (-$8,026, -$5,889) |  |  | -$9,845 | (-$10,998, -$8,691) |  |  |
| Traumatic brain injury |  |  | -$8,835 | (-$11,105, -$6,565) |  |  | -$11,191 | (-$13,459, -$8,924) |
| Internal injury |  |  | -$12,068 | (-$17,132, -$7,003) |  |  | -$8,215 | (-$11,767, -$4,663) |
| Poisoning |  |  | -$6,546 | (-$8,472, -$4,620) |  |  | -$8,189 | (-$10,666, -$5,713) |
| Other injury |  |  | -$4,440 | (-$5,933, -$2,947) |  |  | -$9,539 | (-$11,318, -$7,759) |
| **Neurological conditions** | -$5,848 | (-$7,646, -$4,049) |  |  | -$10,810 | (-$13,336, -$8,283) |  |  |
| Dementia |  |  | -$3,954 | (-$6,128, -$1,780) |  |  | -$8,429 | (-$15,460, -$1,398) |
| Other neurological conditions |  |  | -$7,399 | (-$9,451, -$5,348) |  |  | -$11,672 | (-$14,374, -$8,970) |
| **Respiratory disorders** | -$5,497 | (-$6,627, -$4,368) |  |  | -$6,769 | (-$8,564, -$4,973) |  |  |
| Chronic obstructive pulmonary disease |  |  | -$5,421 | (-$6,710, -$4,133) |  |  | -$6,463 | (-$8,345, -$4,581) |
| Asthma |  |  | -$7,628 | (-$11,523, -$3,733) |  |  | -$10,343 | (-$17,616, -$3,069) |
| Other respiratory disorders |  |  | -$4,697 | (-$7,605, -$1,789) |  |  | -$8,804 | (-$12,902, -$4,706) |
| **Diabetes and other endocrine disorders** | -$6,793 | (-$8,707, -$4,879) |  |  | -$8,298 | (-$10,154, -$6,442) |  |  |
| Type 2 diabetes mellitus |  |  | -$4,797 | (-$6,970, -$2,625) |  |  | -$8,455 | (-$11,140, -$5,770) |
| Other endocrine disorders |  |  | -$7,388 | (-$11,252, -$3,524) |  |  | -$7,692 | (-$10,743, -$4,641) |
| **Gastrointestinal disorders** | -$6,219 | (-$9,410, -$3,028) |  |  | -$7,250 | (-$8,931, -$5,569) |  |  |
| Upper GI disorder |  |  | $2,509 | (-$1,024, $6,042) |  |  | -$1,803 | (-$6,476, $2,869) |
| Chronic liver disease |  |  | -$5,982 | (-$9,628, -$2,335) |  |  | -$8,763 | (-$11,102, -$6,423) |
| Other GI disorders |  |  | -$8,018 | (-$13,933, -$2,104) |  |  | -$4,829 | (-$7,481, -$2,177) |
| **Infections** | -$4,958 | (-$7,128, -$2,789) | -$4,389 | (-$6,545, -$2,233) | -$5,084 | (-$6,936, -$3,232) | -$4,461 | (-$6,299, -$2,624) |
| **Genitourinary disorders** | -$5,865 | (-$9,107, -$2,623) |  |  | -$5,793 | (-$8,638, -$2,948) |  |  |
| Chronic kidney disease |  |  | -$5,427 | (-$8,912, -$1,942) |  |  | -$4,843 | (-$8,365, -$1,321) |
| Other GU disorders |  |  | -$5,331 | (-$11,938, $1,277) |  |  | -$2,777 | (-$7,390, $1,836) |
| **Prevalent years of diagnosis** | | | | | | | | |
| **Cancer** | -$1,971 | (-$2,179, -$1,763) |  |  | -$1,983 | (-$2,358, -$1,608) |  |  |
| Lung |  |  | -$5,379 | (-$6,647, -$4,110) |  |  | -$6,622 | (-$8,981, -$4,263) |
| Colorectal |  |  | -$3,280 | (-$3,996, -$2,564) |  |  | -$3,334 | (-$4,344, -$2,325) |
| Breast |  |  | -$2,336 | (-$2,682, -$1,989) |  |  |  |  |
| Prostate |  |  |  |  |  |  | -$1,490 | (-$2,252, -$727) |
| Other cancer |  |  | -$1,459 | (-$1,737, -$1,182) |  |  | -$1,732 | (-$2,207, -$1,258) |
| **Cardiovascular and blood disorders** | -$1,386 | (-$1,572, -$1,200) |  |  | -$2,228 | (-$2,467, -$1,989) |  |  |
| IHD |  |  | -$1,978 | (-$2,303, -$1,653) |  |  | -$2,171 | (-$2,520, -$1,823) |
| Stroke |  |  | -$4,032 | (-$4,549, -$3,514) |  |  | -$4,892 | (-$5,537, -$4,246) |
| Other CVD |  |  | -$770 | (-$1,007, -$532) |  |  | -$1,423 | (-$1,745, -$1,100) |
| Blood disorders |  |  | -$909 | (-$1,336, -$483) |  |  | -$3,119 | (-$3,925, -$2,313) |
| **Mental illness** | -$5,004 | (-$5,261, -$4,747) |  |  | -$6,097 | (-$6,440, -$5,754) |  |  |
| Anxiety and depressive disorders |  |  | -$4,182 | (-$4,489, -$3,874) |  |  | -$5,212 | (-$5,692, -$4,731) |
| Alcohol use disorders |  |  | -$3,602 | (-$4,220, -$2,985) |  |  | -$4,856 | (-$5,438, -$4,274) |
| Schizophrenia |  |  | -$3,384 | (-$3,945, -$2,823) |  |  | -$4,990 | (-$5,661, -$4,319) |
| Other mental illness |  |  | -$3,048 | (-$3,440, -$2,657) |  |  | -$3,801 | (-$4,303, -$3,299) |
| **Musculoskeletal disorders** | -$1,100 | (-$1,244, -$957) |  |  | -$1,278 | (-$1,461, -$1,094) |  |  |
| Spinal disorders |  |  | -$1,338 | (-$1,642, -$1,034) |  |  | -$2,221 | (-$2,652, -$1,790) |
| Osteoarthritis |  |  | -$1,762 | (-$2,051, -$1,473) |  |  | -$1,132 | (-$1,563, -$700) |
| Chronic musculoskeletal pain syndrome |  |  | -$1,611 | (-$2,003, -$1,219) |  |  | -$2,085 | (-$2,577, -$1,592) |
| Rheumatoid arthritis |  |  | -$3,621 | (-$4,746, -$2,495) |  |  | -$2,621 | (-$5,195, -$48) |
| Other MSK disorders |  |  | -$690 | (-$862, -$517) |  |  | -$833 | (-$1,039, -$626) |
| **Injury** | -$3,475 | (-$5,180, -$1,770) |  |  | -$6,799 | (-$7,954, -$5,644) |  |  |
| Traumatic brain injury |  |  | -$3,629 | (-$5,485, -$1,774) |  |  | -$7,125 | (-$8,421, -$5,828) |
| **Neurological conditions** | -$562 | (-$708, -$415) |  |  | -$1,792 | (-$2,084, -$1,499) |  |  |
| Dementia |  |  | -$7,751 | (-$9,671, -$5,831) |  |  | -$10,589 | (-$13,071, -$8,107) |
| Migraine |  |  | $66 | (-$103, $234) |  |  | -$574 | (-$974, -$174) |
| Primary insomnia |  |  | $600 | (-$2,712, $3,911) |  |  | $372 | (-$5,469, $6,212) |
| Other neurological conditions |  |  | -$2,061 | (-$2,308, -$1,814) |  |  | -$3,095 | (-$3,511, -$2,678) |
| **Respiratory disorders** | -$632 | (-$895, -$368) |  |  | -$585 | (-$959, -$211) |  |  |
| Chronic obstructive pulmonary disease |  |  | -$2,343 | (-$2,875, -$1,811) |  |  | -$3,292 | (-$4,233, -$2,350) |
| Asthma |  |  | -$651 | (-$1,256, -$47) |  |  | -$1,663 | (-$3,007, -$319) |
| Other respiratory disorders |  |  | -$222 | (-$524, $79) |  |  | -$231 | (-$632, $170) |
| **Diabetes and other endocrine disorders** | -$1,972 | (-$2,170, -$1,774) |  |  | -$1,472 | (-$1,768, -$1,177) |  |  |
| Type 2 diabetes mellitus |  |  | -$2,124 | (-$2,331, -$1,918) |  |  | -$1,440 | (-$1,735, -$1,145) |
| Other endocrine disorders |  |  | -$883 | (-$1,308, -$457) |  |  | -$1,234 | (-$2,045, -$423) |
| **Reproductive disorders** | -$567 | (-$715, -$418) | -$590 | (-$739, -$442) |  |  |  |  |
| **Gastrointestinal disorders** | -$787 | (-$936, -$638) |  |  | -$797 | (-$1,007, -$586) |  |  |
| Upper GI disorder |  |  | -$806 | (-$1,100, -$513) |  |  | -$857 | (-$1,304, -$411) |
| Chronic liver disease |  |  | -$3,278 | (-$4,257, -$2,298) |  |  | -$2,867 | (-$3,957, -$1,778) |
| Other GI disorders |  |  | -$643 | (-$801, -$485) |  |  | -$673 | (-$896, -$450) |
| **Genitourinary disorders** | -$28 | (-$320, $263) |  |  | -$938 | (-$1,282, -$595) |  |  |
| Chronic kidney disease |  |  | -$1,022 | (-$1,557, -$488) |  |  | -$602 | (-$1,311, $108) |
| Other GU disorders |  |  | $348 | ($23, $673) |  |  | -$851 | (-$1,216, -$486) |
| **Sensory disorders** | -$1,055 | (-$1,328, -$782) | -$966 | (-$1,238, -$693) | -$1,245 | (-$1,664, -$827) | -$1,192 | (-$1,611, -$774) |
| **Other coefficients** | | | | | | | | |
| **Year** | | | | | | | | |
| 2006 -07 | -$4,187 | (-$4,248, -$4,125) | -$4,257 | (-$4,318, -$4,196) | -$5,624 | (-$5,710, -$5,538) | -$5,709 | (-$5,795, -$5,623) |
| 2007 -08 | -$3,484 | (-$3,540, -$3,427) | -$3,546 | (-$3,603, -$3,489) | -$4,781 | (-$4,860, -$4,702) | -$4,855 | (-$4,934, -$4,775) |
| 2008 -09 | -$3,210 | (-$3,262, -$3,158) | -$3,264 | (-$3,316, -$3,212) | -$4,770 | (-$4,842, -$4,698) | -$4,835 | (-$4,908, -$4,763) |
| 2009 -10 | -$3,293 | (-$3,340, -$3,245) | -$3,340 | (-$3,388, -$3,292) | -$5,634 | (-$5,700, -$5,568) | -$5,692 | (-$5,758, -$5,626) |
| 2010 -11 | -$3,436 | (-$3,479, -$3,393) | -$3,476 | (-$3,520, -$3,433) | -$5,634 | (-$5,694, -$5,574) | -$5,685 | (-$5,744, -$5,625) |
| 2011 -12 | -$3,377 | (-$3,415, -$3,338) | -$3,410 | (-$3,449, -$3,371) | -$5,225 | (-$5,278, -$5,171) | -$5,267 | (-$5,320, -$5,213) |
| 2012 -13 | -$2,817 | (-$2,851, -$2,783) | -$2,843 | (-$2,877, -$2,808) | -$4,122 | (-$4,169, -$4,075) | -$4,156 | (-$4,203, -$4,109) |
| 2013 -14 | -$2,254 | (-$2,282, -$2,225) | -$2,271 | (-$2,300, -$2,242) | -$3,173 | (-$3,213, -$3,133) | -$3,197 | (-$3,237, -$3,157) |
| 2014 -15 | -$1,385 | (-$1,406, -$1,363) | -$1,393 | (-$1,415, -$1,371) | -$1,794 | (-$1,825, -$1,764) | -$1,806 | (-$1,837, -$1,776) |
| 2015 -16 | $0 |  | $0 |  | $0 |  | $0 |  |
| **Deprivation quintile (NZDep)** | | | | | | | | |
| 1 (least deprived) | $10 | (-$49, $70) | $16 | (-$43, $75) | $598 | ($516, $680) | $610 | ($528, $691) |
| 2 | $49 | ($1, $96) | $053 | ($6, $101) | $245 | ($181, $309) | $250 | ($186, $314) |
| 3 (ref) | $0 |  | $0 |  | $0 |  | $0 |  |
| 4 | -$30 | (-$75, $14) | -$037 | (-$81, $7) | -$130 | (-$187, -$074) | -$140 | (-$196, -$083) |
| 5 (most deprived) | -$291 | (-$345, -$237) | -$307 | (-$361, -$253) | -$456 | (-$523, -$389) | -$474 | (-$541, -$408) |
| **Age-group (years)** | | | | | | | | |
| 25 - 29 | -$4,764 | (-$4,898, -$4,630) | -$4,550 | (-$4,684, -$4,416) | -$5,983 | (-$6,170, -$5,796) | -$5,746 | (-$5,933, -$5,559) |
| 30 - 34 | -$4,526 | (-$4,648, -$4,403) | -$4,329 | (-$4,451, -$4,207) | -$2,440 | (-$2,609, -$2,271) | -$2,218 | (-$2,387, -$2,050) |
| 35 - 39 | -$4,469 | (-$4,578, -$4,361) | -$4,288 | (-$4,395, -$4,180) | -$464 | (-$613, -$314) | -$253 | (-$402, -$104) |
| 40 - 44 | -$2,248 | (-$2,332, -$2,164) | -$2,143 | (-$2,226, -$2,059) | $836 | ($716, $955) | $946 | ($828, $1,065) |
| 45 - 49 | -$569 | (-$635, -$503) | -$474 | (-$539, -$409) | $739 | ($646, $832) | $838 | ($746, $929) |
| 50 - 54 | $0 |  | $0 |  | $0 |  | $0 |  |
| 55 - 59 | -$1,514 | (-$1,566, -$1,461) | -$1,497 | (-$1,549, -$1,444) | -$2,118 | (-$2,197, -$2,039) | -$2,101 | (-$2,180, -$2,022) |
| 60 - 64 | -$4,758 | (-$4,846, -$4,669) | -$4,781 | (-$4,868, -$4,693) | -$5,900 | (-$6,037, -$5,763) | -$5,983 | (-$6,118, -$5,848) |
| **Died from other cause** | -$5,020 | (-$6,244, -$3,797) | -$4,656 | (-$5,900, -$3,411) | -$7,005 | (-$8,853, -$5,157) | -$6,756 | (-$8,607, -$4,906) |

† Income loss in year of death not shown for: mental illness due to not being a valid cause of death; subsets of musculoskeletal (as very uncommon); subsets of neurological where death not possible (e.g. migraine, insomnia); and reproductive, skin and sensory disorders.

‡ Prevalent costs not valid for infection, injury and skin disorders (which are treated as acute illnesses).

CVD = cardiovascular disease; IHD = ischaemic heart disease; DM = diabetes mellitus; LLK = lung, liver and kidney disease; CKD = chronic kidney disease; CLD = chronic liver disease; MS = musculoskeletal.
