# Supplementary material for: Disease-related income and economic productivity loss in New Zealand: A longitudinal analysis of linked individual-level data
Source: PLoS Med. 2021 Nov 30;18(11):e1003848. doi: 10.1371/journal.pmed.1003848 (PMC8631646; doi:10.1371/journal.pmed.1003848)
Supplement: S4 Table — (DOCX) [file pmed.1003848.s004.docx]

Supplementary Table 4: Descriptive data (healthy and diseased combined) within observational window 2006 - 07 to 2015 - 16 by sex and age

| **Sex** | **Females** | | | | **Males** | | | |
| --- | --- | --- | --- | --- | --- | --- | --- | --- |
| **Age-group** | **25 - 34** | **35 - 44** | **45 - 54** | **55 - 64** | **25 - 34** | **35 - 44** | **45 - 54** | **55 - 64** |
| Total person-years | 2,786,874 | 3,097,308 | 3,090,582 | 2,449,398 | 2,757,915 | 2,954,421 | 2,984,406 | 2,382,258 |
| Total income in billion (2020 US$) | $56.01 | $68.97 | $74.30 | $45.59 | $80.93 | $108.38 | $104.82 | $67.13 |
| **Person observations by tax year** |  |  |  |  |  |  |  |  |
| 2006 - 07 | 273,411 | 321,537 | 287,571 | 215,625 | 266,319 | 308,916 | 280,401 | 211,428 |
| 2007 - 08 | 272,223 | 320,769 | 296,052 | 223,248 | 265,743 | 307,653 | 287,565 | 218,751 |
| 2008 - 09 | 271,872 | 319,497 | 302,958 | 230,760 | 266,229 | 305,373 | 293,760 | 225,354 |
| 2009 - 10 | 273,264 | 317,982 | 308,247 | 237,972 | 267,930 | 302,865 | 298,587 | 232,149 |
| 2010 - 11 | 273,987 | 314,175 | 311,424 | 244,455 | 268,131 | 298,005 | 300,936 | 237,888 |
| 2011 - 12 | 274,272 | 309,162 | 313,428 | 248,235 | 268,800 | 292,593 | 301,695 | 241,230 |
| 2012 - 13 | 276,849 | 303,933 | 314,757 | 252,486 | 273,750 | 288,165 | 302,406 | 244,797 |
| 2013 - 14 | 282,831 | 299,829 | 317,037 | 258,759 | 283,383 | 285,360 | 305,082 | 250,425 |
| 2014 - 15 | 292,557 | 297,588 | 318,969 | 265,383 | 296,469 | 284,565 | 307,065 | 256,887 |
| 2015 - 16 | 295,605 | 292,836 | 320,139 | 272,478 | 301,164 | 280,932 | 306,912 | 263,346 |
| **Total income in billion (2020 US$)** |  |  |  |  |  |  |  |  |
| 2006 - 07 | $5.46 | $6.58 | $6.46 | $3.40 | $7.87 | $10.85 | $9.35 | $5.60 |
| 2007 - 08 | $5.62 | $6.82 | $6.89 | $3.73 | $8.04 | $11.10 | $9.83 | $5.96 |
| 2008 - 09 | $5.62 | $6.92 | $7.14 | $4.01 | $7.92 | $11.07 | $10.10 | $6.19 |
| 2009 - 10 | $5.46 | $6.89 | $7.28 | $4.28 | $7.58 | $10.71 | $10.05 | $6.26 |
| 2010 - 11 | $5.35 | $6.81 | $7.29 | $4.44 | $7.50 | $10.54 | $10.15 | $6.41 |
| 2011 - 12 | $5.27 | $6.74 | $7.37 | $4.59 | $7.50 | $10.47 | $10.32 | $6.60 |
| 2012 - 13 | $5.38 | $6.81 | $7.58 | $4.86 | $7.86 | $10.62 | $10.67 | $6.94 |
| 2013 - 14 | $5.60 | $6.91 | $7.78 | $5.09 | $8.31 | $10.73 | $11.01 | $7.27 |
| 2014 - 15 | $5.92 | $7.11 | $8.06 | $5.39 | $8.89 | $11.01 | $11.45 | $7.69 |
| 2015 - 16 | $6.33 | $7.37 | $8.48 | $5.81 | $9.46 | $11.28 | $11.89 | $8.21 |
| **Person-year observations by:** |  |  |  |  |  |  |  |  |
| **Ethnicity** |  |  |  |  |  |  |  |  |
| Māori | 492,216 | 477,687 | 416,766 | 254,163 | 478,110 | 461,094 | 398,685 | 238,443 |
| Pacific peoples | 210,489 | 197,313 | 158,841 | 97,440 | 215,973 | 200,043 | 157,662 | 96,312 |
| Asian peoples | 490,350 | 380,334 | 305,289 | 179,061 | 478,758 | 319,908 | 255,000 | 151,449 |
| Other (European) | 1,593,816 | 2,041,971 | 2,209,686 | 1,918,737 | 1,585,074 | 1,973,376 | 2,173,062 | 1,896,051 |
| **Deprivation quintile (NZDep)** |  |  |  |  |  |  |  |  |
| 1 (least deprived) | 421,668 | 677,097 | 749,541 | 588,993 | 400,992 | 595,746 | 706,398 | 582,678 |
| 2 | 522,735 | 647,562 | 661,806 | 527,265 | 507,513 | 599,841 | 630,045 | 511,899 |
| 3 | 580,149 | 615,975 | 599,523 | 484,017 | 571,944 | 595,434 | 576,732 | 464,874 |
| 4 | 623,685 | 587,850 | 552,441 | 450,924 | 630,882 | 587,541 | 544,377 | 430,623 |
| 5 (most deprived) | 638,640 | 568,821 | 527,268 | 398,202 | 646,584 | 575,856 | 526,857 | 392,181 |

All numbers are random rounded to near multiple of 3 as per Statistics New Zealand requirements.
