# Supplementary material for: Disease-related income and economic productivity loss in New Zealand: A longitudinal analysis of linked individual-level data
Source: PLoS Med. 2021 Nov 30;18(11):e1003848. doi: 10.1371/journal.pmed.1003848 (PMC8631646; doi:10.1371/journal.pmed.1003848)
Supplement: S5 Table — (DOCX) [file pmed.1003848.s005.docx]

Supplementary Table 5: Descriptive data (healthy and diseased combined) within observational window 2006 - 07 to 2015 - 16 by sex and ethnicity

| **Sex** | **Females** | | | | **Males** | | | |
| --- | --- | --- | --- | --- | --- | --- | --- | --- |
| **Ethnicity** | **Māori** | **Pacific peoples** | **Asian peoples** | **Other / European** | **Māori** | **Pacific peoples** | **Asian peoples** | **Other / European** |
| Total person-years | 1,640,832 | 664,083 | 1,355,037 | 7,764,213 | 1,576,332 | 669,990 | 1,205,118 | 7,627,563 |
| Total income in billion (2020 US$) | $28.25 | $12.32 | $24.78 | $179.54 | $39.04 | $17.61 | $32.54 | $272.07 |
| **Person observations by tax year** |  |  |  |  |  |  |  |  |
| 2006 - 07 | 156,357 | 61,230 | 102,876 | 777,684 | 150,009 | 61,323 | 88,791 | 766,938 |
| 2007 - 08 | 157,704 | 62,922 | 111,120 | 780,546 | 151,269 | 63,357 | 96,855 | 768,231 |
| 2008 - 09 | 158,886 | 64,512 | 119,109 | 782,574 | 152,115 | 65,001 | 104,856 | 768,744 |
| 2009 - 10 | 161,214 | 65,955 | 125,931 | 784,365 | 154,737 | 66,027 | 110,691 | 770,076 |
| 2010 - 11 | 163,251 | 66,669 | 131,700 | 782,424 | 156,366 | 66,783 | 115,359 | 766,455 |
| 2011 - 12 | 164,028 | 67,035 | 137,910 | 776,124 | 157,032 | 67,284 | 120,867 | 759,129 |
| 2012 - 13 | 164,985 | 67,194 | 144,714 | 771,132 | 158,277 | 67,881 | 128,028 | 754,932 |
| 2013 - 14 | 167,751 | 68,118 | 152,376 | 770,217 | 161,463 | 69,039 | 136,992 | 756,753 |
| 2014 - 15 | 171,255 | 69,660 | 162,342 | 771,234 | 165,330 | 70,992 | 148,839 | 759,825 |
| 2015 - 16 | 175,404 | 70,785 | 166,956 | 767,913 | 169,734 | 72,303 | 153,834 | 756,483 |
| **Total income in billion (2020 US$)** |  |  |  |  |  |  |  |  |
| 2006 - 07 | $2.58 | $1.09 | $1.66 | $16.57 | $3.78 | $1.63 | $2.20 | $26.06 |
| 2007 - 08 | $2.71 | $1.17 | $1.91 | $17.25 | $3.89 | $1.72 | $2.50 | $26.81 |
| 2008 - 09 | $2.78 | $1.21 | $2.11 | $17.58 | $3.88 | $1.74 | $2.74 | $26.92 |
| 2009 - 10 | $2.76 | $1.21 | $2.22 | $17.72 | $3.69 | $1.66 | $2.84 | $26.41 |
| 2010 - 11 | $2.74 | $1.20 | $2.32 | $17.64 | $3.68 | $1.66 | $2.96 | $26.31 |
| 2011 - 12 | $2.73 | $1.20 | $2.43 | $17.61 | $3.68 | $1.67 | $3.12 | $26.44 |
| 2012 - 13 | $2.79 | $1.22 | $2.63 | $17.99 | $3.82 | $1.72 | $3.44 | $27.11 |
| 2013 - 14 | $2.88 | $1.26 | $2.86 | $18.38 | $3.97 | $1.80 | $3.79 | $27.76 |
| 2014 - 15 | $3.04 | $1.33 | $3.14 | $18.97 | $4.21 | $1.94 | $4.23 | $28.66 |
| 2015 - 16 | $3.24 | $1.43 | $3.50 | $19.82 | $4.44 | $2.08 | $4.73 | $29.58 |
| **Person-years observations by:** |  |  |  |  |  |  |  |  |
| **Age-group (years)** |  |  |  |  |  |  |  |  |
| 25 - 34 | 492,216 | 210,489 | 490,350 | 1,593,819 | 478,110 | 215,970 | 478,755 | 1,585,077 |
| 35 - 44 | 477,687 | 197,313 | 380,334 | 2,041,974 | 461,094 | 200,043 | 319,908 | 1,973,376 |
| 45 - 54 | 416,766 | 158,844 | 305,289 | 2,209,683 | 398,685 | 157,659 | 255,000 | 2,173,062 |
| 55 - 64 | 254,163 | 97,437 | 179,061 | 1,918,740 | 238,443 | 96,312 | 151,452 | 1,896,051 |
| **Deprivation quintile (NZDep)** |  |  |  |  |  |  |  |  |
| 1 (least deprived) | 130,671 | 32,166 | 235,065 | 2,039,403 | 128,940 | 33,246 | 185,487 | 1,938,141 |
| 2 | 181,677 | 51,969 | 285,396 | 1,840,326 | 178,095 | 53,853 | 239,646 | 1,777,710 |
| 3 | 255,126 | 76,449 | 297,108 | 1,650,975 | 251,058 | 81,984 | 261,111 | 1,614,834 |
| 4 | 381,486 | 144,186 | 303,318 | 1,385,913 | 370,179 | 144,918 | 285,774 | 1,392,555 |
| 5 (most deprived) | 691,875 | 359,310 | 234,147 | 847,599 | 648,063 | 355,989 | 233,097 | 904,323 |

All numbers are random rounded to near multiple of 3 as per Statistics New Zealand requirements.
