# Supplementary material for: Disease-related income and economic productivity loss in New Zealand: A longitudinal analysis of linked individual-level data
Source: PLoS Med. 2021 Nov 30;18(11):e1003848. doi: 10.1371/journal.pmed.1003848 (PMC8631646; doi:10.1371/journal.pmed.1003848)
Supplement: S6 Table — (DOCX) [file pmed.1003848.s006.docx]

Supplementary Table 6: Descriptive data (healthy and combined) within observational window 2006 - 07 to 2015 - 16 by sex and deprivation

| **Sex** | **Females** | | | | | **Males** | | | | |
| --- | --- | --- | --- | --- | --- | --- | --- | --- | --- | --- |
| **Deprivation** | **1 (least)** | **2** | **3** | **4** | **5 (most)** | **1 (least)** | **2** | **3** | **4** | **5 (most)** |
| Total person-years | 2,437,302 | 2,359,365 | 2,279,661 | 2,214,900 | 2,132,934 | 2,285,814 | 2,249,301 | 2,208,984 | 2,193,426 | 2,141,478 |
| Total income in billion (2020 US$) | $62.59 | $55.71 | $49.62 | $43.82 | $33.14 | $97.76 | $80.65 | $71.00 | $62.87 | $48.99 |
| **Person observations by tax year** |  |  |  |  |  |  |  |  |  |  |
| 2006 - 07 | 233,667 | 225,696 | 218,679 | 215,328 | 204,780 | 221,469 | 214,986 | 211,509 | 212,850 | 206,253 |
| 2007 - 08 | 238,542 | 228,906 | 221,700 | 217,275 | 205,872 | 225,312 | 217,653 | 214,008 | 215,160 | 207,582 |
| 2008 - 09 | 243,645 | 231,411 | 223,671 | 219,306 | 207,051 | 229,629 | 220,644 | 215,379 | 216,246 | 208,815 |
| 2009 - 10 | 248,562 | 234,633 | 226,176 | 221,037 | 207,057 | 233,676 | 223,446 | 217,746 | 218,181 | 208,479 |
| 2010 - 11 | 239,289 | 236,598 | 229,086 | 222,105 | 216,969 | 222,630 | 225,243 | 221,496 | 218,541 | 217,047 |
| 2011 - 12 | 241,017 | 237,153 | 228,741 | 221,427 | 216,762 | 223,785 | 225,327 | 221,190 | 218,208 | 215,808 |
| 2012 - 13 | 242,529 | 237,654 | 229,326 | 221,793 | 216,717 | 225,918 | 225,624 | 222,531 | 219,159 | 215,889 |
| 2013 - 14 | 246,309 | 241,029 | 232,302 | 222,804 | 216,015 | 231,198 | 229,950 | 226,275 | 221,082 | 215,742 |
| 2014 - 15 | 250,560 | 242,832 | 234,561 | 226,602 | 219,939 | 234,927 | 232,992 | 229,086 | 226,188 | 221,787 |
| 2015 - 16 | 253,185 | 243,450 | 235,419 | 227,226 | 221,775 | 237,273 | 233,439 | 229,764 | 227,805 | 224,076 |
| **Total income in billion (2020 US$)** |  |  |  |  |  |  |  |  |  |  |
| 2006 - 07 | $5.48 | $4.93 | $4.47 | $4.00 | $3.02 | $8.92 | $7.41 | $6.61 | $5.95 | $4.78 |
| 2007 - 08 | $5.77 | $5.17 | $4.70 | $4.22 | $3.20 | $9.26 | $7.66 | $6.87 | $6.20 | $4.94 |
| 2008 - 09 | $5.96 | $5.29 | $4.82 | $4.33 | $3.28 | $9.43 | $7.79 | $6.90 | $6.22 | $4.93 |
| 2009 - 10 | $6.11 | $5.36 | $4.87 | $4.33 | $3.23 | $9.44 | $7.71 | $6.79 | $6.04 | $4.62 |
| 2010 - 11 | $6.01 | $5.46 | $4.84 | $4.28 | $3.30 | $9.28 | $7.81 | $6.80 | $5.98 | $4.73 |
| 2011 - 12 | $6.11 | $5.51 | $4.84 | $4.25 | $3.25 | $9.48 | $7.88 | $6.86 | $6.00 | $4.68 |
| 2012 - 13 | $6.35 | $5.66 | $4.97 | $4.35 | $3.30 | $9.84 | $8.12 | $7.11 | $6.23 | $4.80 |
| 2013 - 14 | $6.60 | $5.87 | $5.14 | $4.45 | $3.31 | $10.28 | $8.44 | $7.36 | $6.39 | $4.85 |
| 2014 - 15 | $6.90 | $6.06 | $5.34 | $4.67 | $3.50 | $10.67 | $8.74 | $7.68 | $6.76 | $5.18 |
| 2015 - 16 | $7.31 | $6.39 | $5.63 | $4.93 | $3.73 | $11.15 | $9.09 | $8.01 | $7.10 | $5.48 |
| **Person-years observations by:** |  |  |  |  |  |  |  |  |  |  |
| **Age-group (years)** |  |  |  |  |  |  |  |  |  |  |
| 25 - 34 | 421,668 | 522,735 | 580,146 | 623,685 | 638,637 | 400,992 | 507,516 | 571,944 | 630,882 | 646,581 |
| 35 - 44 | 677,100 | 647,562 | 615,978 | 587,853 | 568,821 | 595,746 | 599,841 | 595,437 | 587,544 | 575,856 |
| 45 - 54 | 749,544 | 661,806 | 599,520 | 552,441 | 527,268 | 706,398 | 630,045 | 576,729 | 544,377 | 526,857 |
| 55 - 64 | 588,990 | 527,265 | 484,014 | 450,924 | 398,205 | 582,678 | 511,902 | 464,874 | 430,623 | 392,181 |
| **Ethnicity** |  |  |  |  |  |  |  |  |  |  |
| Māori | 130,668 | 181,674 | 255,129 | 381,483 | 691,878 | 128,940 | 178,092 | 251,058 | 370,179 | 648,063 |
| Pacific | 32,166 | 51,969 | 76,452 | 144,186 | 359,310 | 33,246 | 53,853 | 81,984 | 144,915 | 355,992 |
| Asian | 235,065 | 285,396 | 297,108 | 303,318 | 234,147 | 185,490 | 239,646 | 261,111 | 285,774 | 233,100 |
| Other | 2,039,400 | 1,840,326 | 1,650,975 | 1,385,913 | 847,599 | 1,938,144 | 1,777,710 | 1,614,834 | 1,392,558 | 904,326 |

All numbers are random rounded to near multiple of 3 as per Statistics New Zealand requirements.
