# Supplementary material for: Disease-related income and economic productivity loss in New Zealand: A longitudinal analysis of linked individual-level data
Source: PLoS Med. 2021 Nov 30;18(11):e1003848. doi: 10.1371/journal.pmed.1003848 (PMC8631646; doi:10.1371/journal.pmed.1003848)
Supplement: S7 Table — (DOCX) [file pmed.1003848.s007.docx]

Supplementary Table 7: Annual income loss (US$ 2020) for 14 and 40 disease models predicted by OLS regression for 50-54 year olds (95% confidence intervals in parentheses)

| **Diseases and conditions** | **Female** | | | | **Male** | | | |
| --- | --- | --- | --- | --- | --- | --- | --- | --- |
|  | **14 Diseases** | | **39 Diseases** | | **13 Diseases** | | **38 Diseases** | |
| Intercept | $29,339 | ($29,271, $29,407) | $29,019 | ($28,952, $29,087) | $40,746 | ($40,651, $40,841) | $40,357 | ($40,263, $40,451) |
| **Year of diagnosis** | | | | | | | | |
| **Cancer** | -$1,254 | (-$1,662, -$845) |  |  | -$950 | (-$1,551, -$350) |  |  |
| Lung |  |  | -$4,359 | (-$6,193, -$2,524) |  |  | -$9,118 | (-$11,925, -$6,311) |
| Colorectal |  |  | -$1,961 | (-$3,324, -$599) |  |  | -$2,871 | (-$4,627, -$1,116) |
| Breast |  |  | -$326 | (-$938, $286) |  |  |  |  |
| Prostate |  |  |  |  |  |  | $2,289 | ($1,259, $3,319) |
| Other cancer |  |  | -$1,699 | (-$2,304, -$1,093) |  |  | -$1,634 | (-$2,463, -$805) |
| **Cardiovascular and blood disorders** | -$1,921 | (-$2,277, -$1,566) |  |  | -$1,400 | (-$1,806, -$993) |  |  |
| IHD |  |  | -$3,244 | (-$3,874, -$2,615) |  |  | -$2,251 | (-$2,840, -$1,663) |
| Stroke |  |  | -$3,288 | (-$4,176, -$2,401) |  |  | -$3,366 | (-$4,406, -$2,327) |
| Other CVD |  |  | -$661 | (-$1,131, -$191) |  |  | $094 | (-$465, $653) |
| Blood disorders |  |  | -$2,722 | (-$3,523, -$1,920) |  |  | -$4,059 | (-$5,494, -$2,625) |
| **Mental illness** | -$10,005 | (-$10,612, -$9,399) |  |  | -$15,234 | (-$16,044, -$14,423) |  |  |
| Anxiety and depressive disorders |  |  | -$8,453 | (-$9,180, -$7,726) |  |  | -$10,439 | (-$11,526, -$9,352) |
| Alcohol use disorders |  |  | -$6,641 | (-$8,043, -$5,240) |  |  | -$12,099 | (-$13,485, -$10,712) |
| Schizophrenia |  |  | -$11,555 | (-$13,281, -$9,829) |  |  | -$16,732 | (-$19,245, -$14,219) |
| Other mental illness |  |  | -$7,695 | (-$8,762, -$6,628) |  |  | -$14,481 | (-$15,897, -$13,064) |
| **Musculoskeletal disorders** | -$425 | (-$695, -$156) |  |  | -$1,215 | (-$1,527, -$902) |  |  |
| Spinal disorders |  |  | -$2,632 | (-$3,263, -$2,001) |  |  | -$4,976 | (-$5,831, -$4,122) |
| Osteoarthritis |  |  | -$1,961 | (-$2,500, -$1,422) |  |  | -$3,847 | (-$4,587, -$3,107) |
| Chronic musculoskeletal pain syndrome |  |  | -$2,666 | (-$3,466, -$1,867) |  |  | -$4,388 | (-$5,302, -$3,474) |
| Rheumatoid arthritis |  |  | -$4,105 | (-$6,721, -$1,488) |  |  | -$5,068 | (-$10,301, $164) |
| Other MSK disorders |  |  | $441 | ($122, $760) |  |  | -$093 | (-$439, $252) |
| **Injury** | -$2,816 | (-$3,070, -$2,561) |  |  | -$5,840 | (-$6,152, -$5,527) |  |  |
| Traumatic brain injury |  |  | -$5,386 | (-$8,436, -$2,336) |  |  | -$6,280 | (-$8,971, -$3,590) |
| Internal injury |  |  | -$4,293 | (-$7,123, -$1,462) |  |  | -$2,094 | (-$4,235, $047) |
| Poisoning |  |  | -$2,936 | (-$3,719, -$2,154) |  |  | -$4,481 | (-$5,748, -$3,213) |
| Other injury |  |  | -$2,168 | (-$2,436, -$1,900) |  |  | -$5,473 | (-$5,796, -$5,150) |
| **Neurological conditions** | -$825 | (-$1,130, -$520) |  |  | -$3,247 | (-$3,826, -$2,668) |  |  |
| Dementia |  |  | -$12,300 | (-$15,129, -$9,470) |  |  | -$16,708 | (-$20,423, -$12,992) |
| Migraine |  |  | $886 | ($533, $1,240) |  |  | $415 | (-$369, $1,200) |
| Primary insomnia |  |  | -$3,877 | (-$11,545, $3,791) |  |  | -$6,037 | (-$17,151, $5,077) |
| Other neurological conditions |  |  | -$3,933 | (-$4,448, -$3,419) |  |  | -$5,666 | (-$6,479, -$4,852) |
| **Respiratory disorders** | -$655 | (-$1,178, -$133) |  |  | -$062 | (-$744, $619) |  |  |
| Chronic obstructive pulmonary disease |  |  | -$5,692 | (-$6,811, -$4,572) |  |  | -$8,294 | (-$10,070, -$6,518) |
| Asthma |  |  | -$1,546 | (-$2,954, -$138) |  |  | -$2,070 | (-$4,803, $662) |
| Other respiratory disorders |  |  | $330 | (-$262, $923) |  |  | $1,155 | ($424, $1,887) |
| **Diabetes and other endocrine disorders** | -$1,934 | (-$2,308, -$1,560) |  |  | -$1,083 | (-$1,584, -$583) |  |  |
| Type 2 diabetes mellitus |  |  | -$2,173 | (-$2,567, -$1,779) |  |  | -$968 | (-$1,464, -$472) |
| Other endocrine disorders |  |  | -$666 | (-$1,502, $169) |  |  | -$1,117 | (-$2,701, $466) |
| **Reproductive disorders** | $625 | ($318, $932) | $511 | ($204, $818) |  |  |  |  |
| **Gastrointestinal disorders** | -$052 | (-$336, $232) |  |  | $136 | (-$232, $504) |  |  |
| Upper GI disorder |  |  | -$1,494 | (-$2,110, -$878) |  |  | -$3,518 | (-$4,415, -$2,622) |
| Chronic liver disease |  |  | -$4,331 | (-$5,994, -$2,668) |  |  | -$7,092 | (-$9,159, -$5,025) |
| Other GI disorders |  |  | $238 | (-$063, $539) |  |  | $678 | ($293, $1,063) |
| **Infections** | -$2,858 | (-$3,150, -$2,566) | -$2,336 | (-$2,629, -$2,044) | -$4,525 | (-$4,931, -$4,120) | -$3,827 | (-$4,233, -$3,420) |
| **Genitourinary disorders** | -$806 | (-$1,389, -$223) |  |  | $416 | (-$195, $1,028) |  |  |
| Chronic kidney disease |  |  | -$1,108 | (-$2,137, -$078) |  |  | -$1,495 | (-$2,739, -$250) |
| Other GU disorders |  |  | -$786 | (-$1,443, -$129) |  |  | $551 | (-$102, $1,203) |
| **Skin disorders** | -$251 | (-$836, $333) | -$160 | (-$744, $423) | -$959 | (-$1,827, -$092) | -$874 | (-$1,740, -$007) |
| **Sensory disorders** | -$1,305 | (-$1,824, -$785) | -$1,248 | (-$1,767, -$728) | -$1,575 | (-$2,319, -$830) | -$1,572 | (-$2,316, -$829) |
| **Last year of life if died from disease** | | | | | | | | |
| **Cancer** | -$15,431 | (-$16,159, -$14,703) |  |  | -$19,582 | (-$20,634, -$18,530) |  |  |
| Lung |  |  | -$14,080 | (-$15,770, -$12,390) |  |  | -$20,234 | (-$22,706, -$17,763) |
| Colorectal |  |  | -$15,798 | (-$18,071, -$13,525) |  |  | -$19,876 | (-$22,703, -$17,050) |
| Breast |  |  | -$15,674 | (-$17,120, -$14,229) |  |  |  |  |
| Prostate |  |  |  |  |  |  | -$20,744 | (-$26,317, -$15,171) |
| Other cancer |  |  | -$15,133 | (-$16,214, -$14,053) |  |  | -$18,589 | (-$19,902, -$17,276) |
| **Cardiovascular and blood disorders** | -$12,928 | (-$14,419, -$11,436) |  |  | -$17,273 | (-$18,530, -$16,016) |  |  |
| IHD |  |  | -$10,529 | (-$12,926, -$8,131) |  |  | -$15,906 | (-$17,429, -$14,384) |
| Stroke |  |  | -$12,320 | (-$14,975, -$9,665) |  |  | -$16,808 | (-$20,554, -$13,061) |
| Other CVD |  |  | -$11,488 | (-$14,321, -$8,655) |  |  | -$17,384 | (-$20,196, -$14,572) |
| Blood disorders |  |  | -$11,469 | (-$22,347, -$590) |  |  | -$15,482 | (-$30,463, -$501) |
| **Musculoskeletal disorders** | -$15,334 | (-$22,058, -$8,611) |  |  | -$21,650 | (-$37,358, -$5,942) |  |  |
| Other MSK disorders |  |  | -$13,884 | (-$20,601, -$7,166) |  |  | -$17,924 | (-$33,617, -$2,231) |
| **Injury** | -$12,248 | (-$14,271, -$10,225) |  |  | -$17,277 | (-$19,002, -$15,553) |  |  |
| Traumatic brain injury |  |  | -$12,099 | (-$16,335, -$7,863) |  |  | -$17,485 | (-$20,662, -$14,308) |
| Internal injury |  |  | -$10,936 | (-$18,446, -$3,427) |  |  | -$13,212 | (-$19,015, -$7,408) |
| Poisoning |  |  | -$10,446 | (-$13,985, -$6,908) |  |  | -$17,647 | (-$21,685, -$13,609) |
| Other injury |  |  | -$11,007 | (-$14,346, -$7,667) |  |  | -$16,213 | (-$18,847, -$13,580) |
| **Neurological conditions** | -$17,571 | (-$20,491, -$14,651) |  |  | -$26,592 | (-$30,424, -$22,760) |  |  |
| Dementia |  |  | -$18,982 | (-$26,629, -$11,335) |  |  | -$25,545 | (-$38,176, -$12,914) |
| Other neurological conditions |  |  | -$17,047 | (-$20,209, -$13,884) |  |  | -$25,639 | (-$29,662, -$21,617) |
| **Respiratory disorders** | -$15,333 | (-$17,897, -$12,768) |  |  | -$20,191 | (-$23,945, -$16,436) |  |  |
| Chronic obstructive pulmonary disease |  |  | -$14,144 | (-$17,170, -$11,117) |  |  | -$20,534 | (-$25,182, -$15,885) |
| Asthma |  |  | -$13,865 | (-$22,005, -$5,724) |  |  | -$23,402 | (-$41,172, -$5,631) |
| Other respiratory disorders |  |  | -$11,190 | (-$17,358, -$5,023) |  |  | -$15,193 | (-$22,367, -$8,018) |
| **Diabetes and other endocrine disorders** | -$12,409 | (-$16,019, -$8,799) |  |  | -$17,326 | (-$20,962, -$13,691) |  |  |
| Type 2 diabetes mellitus |  |  | -$10,583 | (-$15,702, -$5,463) |  |  | -$16,573 | (-$21,694, -$11,453) |
| Other endocrine disorders |  |  | -$7,647 | (-$13,398, -$1,897) |  |  | -$13,445 | (-$19,020, -$7,870) |
| **Gastrointestinal disorders** | -$12,644 | (-$16,203, -$9,085) |  |  | -$18,058 | (-$21,817, -$14,300) |  |  |
| Upper GI disorder |  |  | -$3,974 | (-$18,317, $10,369) |  |  | -$16,300 | (-$30,245, -$2,356) |
| Chronic liver disease |  |  | -$13,249 | (-$18,174, -$8,324) |  |  | -$18,409 | (-$23,331, -$13,488) |
| Other GI disorders |  |  | -$12,368 | (-$18,017, -$6,719) |  |  | -$13,188 | (-$19,747, -$6,629) |
| **Infections** | -$12,278 | (-$16,337, -$8,218) | -$10,334 | (-$14,391, -$6,278) | -$20,168 | (-$24,527, -$15,809) | -$17,469 | (-$21,827, -$13,110) |
| **Genitourinary disorders** | -$13,592 | (-$21,785, -$5,399) |  |  | -$17,742 | (-$25,043, -$10,441) |  |  |
| Chronic kidney disease |  |  | -$9,819 | (-$19,071, -$566) |  |  | -$14,426 | (-$22,441, -$6,411) |
| Other GU disorders |  |  | -$13,808 | (-$35,526, $7,911) |  |  | -$18,327 | (-$36,790, $136) |
| **Prevalent years of diagnosis** | | | | | | | | |
| **Cancer** | -$304 | (-$465, -$143) |  |  | -$606 | (-$901, -$311) |  |  |
| Lung |  |  | -$6,460 | (-$8,071, -$4,849) |  |  | -$10,990 | (-$13,816, -$8,165) |
| Colorectal |  |  | -$1,139 | (-$1,844, -$434) |  |  | -$1,240 | (-$2,175, -$304) |
| Breast |  |  | $242 | ($026, $458) |  |  |  |  |
| Prostate |  |  |  |  |  |  | $2,038 | ($1,481, $2,595) |
| Other cancer |  |  | -$931 | (-$1,181, -$681) |  |  | -$1,474 | (-$1,844, -$1,104) |
| **Cardiovascular and blood disorders** | -$1,838 | (-$1,934, -$1,743) |  |  | -$1,413 | (-$1,546, -$1,279) |  |  |
| IHD |  |  | -$2,125 | (-$2,350, -$1,900) |  |  | -$2,176 | (-$2,396, -$1,957) |
| Stroke |  |  | -$3,142 | (-$3,451, -$2,833) |  |  | -$3,805 | (-$4,203, -$3,406) |
| Other CVD |  |  | -$887 | (-$1,001, -$774) |  |  | -$041 | (-$201, $118) |
| Blood disorders |  |  | -$1,734 | (-$1,902, -$1,566) |  |  | -$2,441 | (-$2,801, -$2,081) |
| **Mental illness** | -$9,671 | (-$9,797, -$9,545) |  |  | -$15,398 | (-$15,574, -$15,223) |  |  |
| Anxiety and depressive disorders |  |  | -$5,892 | (-$6,054, -$5,730) |  |  | -$7,016 | (-$7,279, -$6,752) |
| Alcohol use disorders |  |  | -$4,217 | (-$4,514, -$3,920) |  |  | -$7,953 | (-$8,250, -$7,656) |
| Schizophrenia |  |  | -$10,017 | (-$10,353, -$9,681) |  |  | -$16,406 | (-$16,851, -$15,960) |
| Other mental illness |  |  | -$5,599 | (-$5,819, -$5,380) |  |  | -$10,906 | (-$11,204, -$10,609) |
| **Musculoskeletal disorders** | -$709 | (-$797, -$621) |  |  | -$1,461 | (-$1,567, -$1,356) |  |  |
| Spinal disorders |  |  | -$2,065 | (-$2,236, -$1,894) |  |  | -$4,023 | (-$4,256, -$3,791) |
| Osteoarthritis |  |  | -$1,392 | (-$1,628, -$1,156) |  |  | -$2,379 | (-$2,685, -$2,073) |
| Chronic musculoskeletal pain syndrome |  |  | -$1,551 | (-$1,803, -$1,300) |  |  | -$2,326 | (-$2,627, -$2,024) |
| Rheumatoid arthritis |  |  | -$3,732 | (-$4,291, -$3,174) |  |  | -$4,809 | (-$6,067, -$3,552) |
| Other MSK disorders |  |  | $267 | ($164, $370) |  |  | -$420 | (-$536, -$304) |
| **Injury** | -$5,082 | (-$5,889, -$4,275) |  |  | -$9,383 | (-$10,002, -$8,764) |  |  |
| Traumatic brain injury |  |  | -$4,175 | (-$4,982, -$3,369) |  |  | -$8,507 | (-$9,127, -$7,887) |
| **Neurological conditions** | -$1,623 | (-$1,715, -$1,531) |  |  | -$5,131 | (-$5,296, -$4,965) |  |  |
| Dementia |  |  | -$7,408 | (-$8,542, -$6,275) |  |  | -$9,669 | (-$11,150, -$8,188) |
| Migraine |  |  | $698 | ($583, $812) |  |  | -$180 | (-$454, $094) |
| Primary insomnia |  |  | $1,973 | ($228, $3,719) |  |  | -$1,719 | (-$4,195, $758) |
| Other neurological conditions |  |  | -$3,917 | (-$4,048, -$3,786) |  |  | -$6,374 | (-$6,574, -$6,174) |
| **Respiratory disorders** | -$887 | (-$1,009, -$765) |  |  | -$1,187 | (-$1,353, -$1,020) |  |  |
| Chronic obstructive pulmonary disease |  |  | -$4,415 | (-$4,779, -$4,051) |  |  | -$5,827 | (-$6,406, -$5,248) |
| Asthma |  |  | -$194 | (-$393, $006) |  |  | -$1,891 | (-$2,273, -$1,510) |
| Other respiratory disorders |  |  | -$106 | (-$254, $041) |  |  | -$180 | (-$362, $003) |
| **Diabetes and other endocrine disorders** | -$2,247 | (-$2,361, -$2,134) |  |  | -$1,393 | (-$1,550, -$1,235) |  |  |
| Type 2 diabetes mellitus |  |  | -$2,280 | (-$2,420, -$2,139) |  |  | -$1,332 | (-$1,517, -$1,148) |
| Other endocrine disorders |  |  | -$897 | (-$1,059, -$734) |  |  | -$404 | (-$650, -$159) |
| **Reproductive disorders** | -$209 | (-$279, -$140) | -$266 | (-$336, -$197) |  |  |  |  |
| **Gastrointestinal disorders** | -$1,000 | (-$1,081, -$918) |  |  | -$1,236 | (-$1,348, -$1,125) |  |  |
| Upper GI disorder |  |  | -$1,879 | (-$2,045, -$1,712) |  |  | -$2,696 | (-$2,917, -$2,476) |
| Chronic liver disease |  |  | -$1,978 | (-$2,383, -$1,574) |  |  | -$3,952 | (-$4,433, -$3,471) |
| Other GI disorders |  |  | -$523 | (-$609, -$436) |  |  | -$514 | (-$633, -$395) |
| **Genitourinary disorders** | -$1,276 | (-$1,420, -$1,132) |  |  | -$642 | (-$822, -$463) |  |  |
| Chronic kidney disease |  |  | -$1,032 | (-$1,308, -$755) |  |  | -$814 | (-$1,222, -$407) |
| Other GU disorders |  |  | -$1,063 | (-$1,223, -$904) |  |  | -$439 | (-$631, -$248) |
| **Sensory disorders** | -$1,557 | (-$1,717, -$1,398) | -$1,204 | (-$1,363, -$1,044) | -$2,536 | (-$2,756, -$2,315) | -$2,288 | (-$2,509, -$2,068) |
| **Other coefficients** | | | | | | | | |
| **Year** | | | | | | | | |
| 2006 -07 | -$4,623 | (-$4,685, -$4,561) | -$4,429 | (-$4,491, -$4,367) | -$5,620 | (-$5,708, -$5,532) | -$5,362 | (-$5,450, -$5,275) |
| 2007 -08 | -$3,758 | (-$3,820, -$3,697) | -$3,583 | (-$3,645, -$3,521) | -$4,655 | (-$4,743, -$4,568) | -$4,423 | (-$4,511, -$4,336) |
| 2008 -09 | -$3,344 | (-$3,406, -$3,283) | -$3,191 | (-$3,253, -$3,130) | -$4,489 | (-$4,577, -$4,402) | -$4,288 | (-$4,375, -$4,201) |
| 2009 -10 | -$3,297 | (-$3,358, -$3,236) | -$3,168 | (-$3,229, -$3,107) | -$5,253 | (-$5,339, -$5,166) | -$5,081 | (-$5,168, -$4,995) |
| 2010 -11 | -$3,270 | (-$3,331, -$3,209) | -$3,167 | (-$3,228, -$3,106) | -$5,013 | (-$5,099, -$4,926) | -$4,876 | (-$4,962, -$4,789) |
| 2011 -12 | -$3,146 | (-$3,207, -$3,085) | -$3,065 | (-$3,126, -$3,004) | -$4,567 | (-$4,654, -$4,481) | -$4,462 | (-$4,548, -$4,375) |
| 2012 -13 | -$2,539 | (-$2,600, -$2,478) | -$2,482 | (-$2,543, -$2,421) | -$3,459 | (-$3,545, -$3,372) | -$3,386 | (-$3,473, -$3,300) |
| 2013 -14 | -$2,007 | (-$2,068, -$1,946) | -$1,971 | (-$2,032, -$1,910) | -$2,661 | (-$2,747, -$2,575) | -$2,615 | (-$2,701, -$2,528) |
| 2014 -15 | -$1,257 | (-$1,317, -$1,196) | -$1,238 | (-$1,298, -$1,177) | -$1,530 | (-$1,616, -$1,444) | -$1,507 | (-$1,593, -$1,421) |
| 2015 -16 | $0 |  | $0 |  | $0 |  | $0 |  |
| **Ethnicity** | | | | | | | | |
| Māori | -$3,751 | (-$3,807, -$3,695) | -$3,754 | (-$3,809, -$3,699) | -$7,228 | (-$7,306, -$7,151) | -$7,260 | (-$7,336, -$7,184) |
| Pacific | -$2,568 | (-$2,646, -$2,489) | -$2,428 | (-$2,506, -$2,351) | -$5,530 | (-$5,637, -$5,422) | -$5,368 | (-$5,474, -$5,261) |
| Asian | -$6,012 | (-$6,063, -$5,960) | -$5,838 | (-$5,889, -$5,787) | -$9,469 | (-$9,543, -$9,395) | -$9,246 | (-$9,319, -$9,172) |
| Other (ref) | $0 |  | $0 |  | $0 |  | $0 |  |
| **Deprivation quintile (NZDep)** | | | | | | | | |
| 1 (least deprived) | $2,919 | ($2,873, $2,965) | $2,963 | ($2,917, $3,008) | $9,395 | ($9,330, $9,460) | $9,447 | ($9,383, $9,512) |
| 2 | $1,340 | ($1,296, $1,384) | $1,369 | ($1,325, $1,413) | $3,082 | ($3,020, $3,145) | $3,116 | ($3,054, $3,179) |
| 3 (ref) | $0 |  | $0 |  | $0 |  | $0 |  |
| 4 | -$1,299 | (-$1,344, -$1,254) | -$1,335 | (-$1,380, -$1,290) | -$2,495 | (-$2,558, -$2,432) | -$2,541 | (-$2,604, -$2,478) |
| 5 (most deprived) | -$4,591 | (-$4,641, -$4,541) | -$4,662 | (-$4,712, -$4,612) | -$6,730 | (-$6,800, -$6,661) | -$6,843 | (-$6,912, -$6,774) |
| **Age-group (years)** | | | | | | | | |
| 25 - 29 | -$3,348 | (-$3,410, -$3,286) | -$3,333 | (-$3,395, -$3,271) | -$5,149 | (-$5,235, -$5,062) | -$5,147 | (-$5,233, -$5,061) |
| 30 - 34 | -$1,859 | (-$1,922, -$1,797) | -$1,809 | (-$1,871, -$1,747) | $437 | ($350, $524) | $497 | ($411, $583) |
| 35 - 39 | -$1,585 | (-$1,647, -$1,524) | -$1,513 | (-$1,574, -$1,451) | $3,381 | ($3,295, $3,467) | $3,473 | ($3,388, $3,558) |
| 40 - 44 | -$583 | (-$644, -$521) | -$580 | (-$641, -$519) | $4,186 | ($4,101, $4,271) | $4,135 | ($4,051, $4,220) |
| 45 - 49 | $464 | ($401, $526) | $511 | ($449, $573) | $2,846 | ($2,760, $2,932) | $2,832 | ($2,746, $2,917) |
| 50 - 54 | $0 |  | $0 |  | $0 |  | $0 |  |
| 55 - 59 | -$2,666 | (-$2,722, -$2,610) | -$2,598 | (-$2,654, -$2,542) | -$3,431 | (-$3,511, -$3,352) | -$3,390 | (-$3,469, -$3,310) |
| 60 - 64 | -$8,836 | (-$8,911, -$8,761) | -$8,741 | (-$8,814, -$8,667) | -$9,782 | (-$9,889, -$9,676) | -$9,677 | (-$9,782, -$9,573) |
| **Died from other cause** | -$16,837 | (-$19,275, -$14,399) | -$15,508 | (-$17,944, -$13,072) | -$19,821 | (-$23,025, -$16,617) | -$19,059 | (-$22,260, -$15,858) |
