# Supplementary material for: Disease-related income and economic productivity loss in New Zealand: A longitudinal analysis of linked individual-level data
Source: PLoS Med. 2021 Nov 30;18(11):e1003848. doi: 10.1371/journal.pmed.1003848 (PMC8631646; doi:10.1371/journal.pmed.1003848)
Supplement: S1 Fig — COPD, chronic obstructive pulmonary disease; CVD, cardiovascular disease; FE, fixed effects; GI, gastrointestinal; GU, genitourinary; MSK, musculoskeletal; OLS, ordinary least squares; TBI, traumatic brain injury; T2DM, type 2 diabetes mellitus. (DOCX) [file pmed.1003848.s008.docx]

Supplementary Figure 1: Annual income loss by disease phase for three model specifications: FE (main analysis); OLS unadjusted for prior income; OLS adjusted for prior income and restricted to health people at beginning of observation window

Both OLS regression models were adjusted for age (five-year age-groups), deprivation (quintile), ethnicity (Asian, Māori, Pacific, Other (European)), and interactions of same with disease phase. The model adjusted for prior income used average income in years in the two-year period 2003-04 to 2004-05. (i.e. prior to observation window), for observations restricted to people with no disease leading into the observation window).
