# Supplementary material for: Disease-related income and economic productivity loss in New Zealand: A longitudinal analysis of linked individual-level data
Source: PLoS Med. 2021 Nov 30;18(11):e1003848. doi: 10.1371/journal.pmed.1003848 (PMC8631646; doi:10.1371/journal.pmed.1003848)
Supplement: S2 Fig — COPD, chronic obstructive pulmonary disease; CVD, cardiovascular disease; FE, fixed effects; GI, gastrointestinal; GU, genitourinary; MSK, musculoskeletal; TBI, traumatic brain injury; T2DM, type 2 diabetes mellitus. (DOCX) [file pmed.1003848.s009.docx]

Supplementary Figure 2: Comparison of income within-individual income change for year before diagnosis and first year of diagnosis, fixed effects model

1. **Female**

1. **Male**
