# Supplementary material for: Disease-related income and economic productivity loss in New Zealand: A longitudinal analysis of linked individual-level data
Source: PLoS Med. 2021 Nov 30;18(11):e1003848. doi: 10.1371/journal.pmed.1003848 (PMC8631646; doi:10.1371/journal.pmed.1003848)
Supplement: S3 Fig — (DOCX) [file pmed.1003848.s010.docx]

Supplementary Figure 3: Proportionate contribution of diseases to human capital approach productivity loss in Norway in 2013 (source (Kinge et al., 2017))
