# Supplementary material for: Disease-related income and economic productivity loss in New Zealand: A longitudinal analysis of linked individual-level data
Source: PLoS Med. 2021 Nov 30;18(11):e1003848. doi: 10.1371/journal.pmed.1003848 (PMC8631646; doi:10.1371/journal.pmed.1003848)
Supplement: S4 Fig — CVD, cardiovascular disease; DALY, disability-adjusted life year; IHD, ischaemic heart disease; YLD, years lost to disability. (DOCX) [file pmed.1003848.s011.docx]

Supplementary Figure 4: Cause-deleted income gain, plotted against YLDs and DALYs in 2011 from the GBD for 25-64 year olds in NZ

YLDs and YLLs sourced from GBD for 2011, using <http://ghdx.healthdata.org/>
