# Supplementary material for: Disease-related income and economic productivity loss in New Zealand: A longitudinal analysis of linked individual-level data
Source: PLoS Med. 2021 Nov 30;18(11):e1003848. doi: 10.1371/journal.pmed.1003848 (PMC8631646; doi:10.1371/journal.pmed.1003848)
Supplement: Attachment — Submitted filename: Plos Med Income Loss Revision Letter.docx [file pmed.1003848.s013.docx]

Dear Editor

20 Oct 2020

**Re:	Disease-related income and economic productivity loss: longitudinal analysis of individual-level data for an entire country (PMEDICINE-D-20-03416R1)**

**REQUESTS FROM THE EDITORS:**

*At this stage, we ask that you include a short, **non-technical Author Summary** of your research to make findings accessible to a wide audience that includes both scientists and non-scientists. The Author Summary should immediately follow the Abstract in your revised manuscript. This text is subject to editorial change and should be distinct from the scientific abstract. Please see our author guidelines for more information: <https://journals.plos.org/plosmedicine/s/revising-your-manuscript#loc-author-summary>

*Author response: Done.*

*Please clarify whether the analytical approach reported here corresponded to one laid out in a prospective protocol or analysis plan? Please state this (either way) early in the Methods section.

*Author response: We have inserted these two sentences immediately beneath the “Analyses” sub-heading in the Methods:*

*“Our analytical plan for this study was to follow the analyses we previously conducted (and published in this Journal) for disease-related health system expenditure. We however deviated from this previous approach to use fixed effects for the ‘main analyses’, reporting OLS regression in sensitivity analyses due to concerns about residual confounding (below).”*

a) If a prospective analysis plan (from your funding proposal, IRB or other ethics committee submission, study protocol, or other planning document written before analyzing the data) was used in designing the study, please include the relevant prospectively written document with your revised manuscript as a Supporting Information file to be published alongside your study, and cite it in the Methods section. A legend for this file should be included at the end of your manuscript.

b) If no such document exists, please make sure that the Methods section transparently describes when analyses were planned, and when/why any data-driven changes to analyses took place.

c) In either case, changes in the analysis-- including those made in response to peer review comments-- should be identified as such in the Methods section of the paper, with rationale.

*Author response: Please see above.*

*We would suggest checking whether any established reporting guideline has been developed relevant to this study type, if so we would suggest using that to support reporting of the study - the RECORD guideline may be relevant potentially and could be worth looking at (<https://www.equator-network.org/reporting-guidelines/record/>) - this is designed for studies done using routinely-collected health data. If the authors feel this is relevant please add the completed RECORD checklist as a supporting information file alongside the revised paper.

*Author response: We have included the RECORD Checklist as a Supplementary File.*

-----------------------------------------------------------

Comments from the reviewers:

Reviewer #1: I confine my remarks to statistical aspects of this paper. I have some concerns that need to be addressed before I can recommend publication

The biggest one is whether the fixed effect regression is the appropriate method. More detail is needed about the method in the 1st para of p. 6. My preference would be for a multilevel model, but the methods used may be OK --it depends on details.

*Author response: Fixed effects is a standard econometric method for analyzing panel data, and removes all time-invariant confounding. (Random effects or multilevel models generate a weighted estimate of the between and within-person association, and hence can be residually confounded by time invariant confounders.[1]) For instance, Jacobson et al., (1993) [2] and Zajacova et al., (2015)[3] have used fixed effect regression for estimating the income/earning loss (For detail see (Jacobson et al., 1993)).* *FE requires less assumption of the data; incorrectly using RE when the assumption for RE are not met, will mean the estimates are biased. We have amended the opening sentence under the sub-heading “Fixed effects regression modelling”:*

*“We used an ‘excess’ or ‘net’ cost approach [4-7] [8], with total income as the dependent variable in ordinary least squares (OLS) fixed effects (FE) regression analyses (uses within individual variation that removes time invariant confounding [1] with cluster-adjusted standard errors).*

Another general concern is what population you are inferrring to. You have the whole population of New Zealand. Are you inferring to the rest of the world? That seems likely to be very wrong. Or perhaps to other high-income countries? Or what? Some statisticians justify this by inferrring to some "super-population" -- I am not a big fan of this, but it's not outright wrong. But you need to specify what the population or super population is.

*Author response: We are inferring to the total NZ population, then talking about generalizability in the Discussion (see below).*

The authors also categorized many continuous variables. This is nearly always a mistake. In *Regression Modeling Strategies* Frank Harrell lists 11 problems with this and sums up "nothing could be more disastrous". I wrote a blog post showing some of the problems, graphically <https://medium.com/@peterflom/what-happens-when-we-categorize-an-independent-variable-in-regression-77d4c5862b6c> Instead, leave the variables continuous and use splines to investigate nonlinearity.

*Author response: Thank you for this comment. We tried extensive alternative specification of variables in analyses prior to submission. Continuous variants for age (say) were problematic due to non-linearities by age, requiring separate specification by each disease in interaction terms and such like. We had problems with (say) quadratics for age generating predictions that were far from the observed data at the younger and older age groups. For model parsimony reasons we used categorical variables. We note that with the entire population of NZ included, that study power is good (i.e. we were not sacrificing many degrees of freedom). Second, we note that with covariate adjustment in epidemiology and biostatistics, it is common practice to use categorical variables – with a common rule of thumb being that more than 4 or 5 categories captures the majority of confounding.[9] Third, we note that with cause deleted analyses categorical variables are perhaps a bit easier conceptually.*

*We have a lot of empathy with this referee’s comment* ***IF*** *the objective of the research is to look at one exposure in particular that can be conceptualized and specified continuously. For example, if we were looking at the association of sodium (exposure) with blood pressure (outcome), then using (say) cubic splines or fractional polynomials is critical given theoretical and prior research about a non-linear association of sodium with blood pressure.* ***But that is not what we are doing.***

*What we are doing is looking at the association of multiple exposures simultaneously (i.e. diseases) with income loss. The diseases (exposures) are* ***genuinely*** *nominal variables: no diagnosis, first year of diagnosis, prevalent, last year of diagnosis if dying of that disease. The covariates that could be considered as continuous are only age and deprivation – and they are ‘only’ covariates. As above, we actually trialed continuous and quadratic specifications of age – it was very cumbersome given interaction terms, and (certainly) not parsimonious for out objective focused on diseases (and then using cause-deleted). For deprivation, we did use categorical quintiles as main effect – and a simple continuous integer variable in interactions (coded 0, 1, 2, 3, 4).*

*We stand by our approach.*

Finally, the figures need work Figure 1 is a dynamite plot. These are not recommended. See e,g, <https://simplystatistics.org/2019/02/21/dynamite-plots-must-die/>

*We are disappointed the reviewer did not like Figure 1 – we spent a lot of time on this!*

*Nevertheless, we note the blog Peter Flom refers to above. It seems to speak (usefully) to lab science more where smaller sample sizes are in use. And the analyses are more descriptive.*

*We are presenting many results from fixed effects regressions, with their 95% confidence interval (not the SD). This is standard practice in our primary field of epidemiology.*

*We note that we did originally have tables, but they were too time consuming to interpret – hence the graphs we present which are more reader friendly. We also assist the reader by placing the central estimate on as a label (CI values are in the Supplementary Table 3). Again, we think this is parsimonious data presentation.*

*We considered the box plots from the blog – thank you. But they seem more for descriptive data with percentile presentation; we have coefficients from a fixed effects regression with 95% CI.*

*So, we think that on balance the current graphs are best, but would accept a counter decision by the Editor to redo them (and please suggest what the better alternative is).*

Figure 2 is a stacked bar chart. These also cannot be recommended, see the work of William S. Cleveland, who showed that people cannot estimate the size of the bars. A Cleveland dot plot might be better, or perhaps a mosaic plot.

*Thank you for the suggestion of a Cleveland dot plot. This appear to be for single estimates with their uncertainty; we have multiple mutually exclusive components of the one total number.*

*We note we published similar stacked plots with the predecessor paper to this one (in Plos Med)[8], and the same type of graph is used routinely in GBD outputs (i.e. Lancet papers).*

Lastly, fig. 3 is a pie chart, also not a good graphic. See my article: Graphics for univariate data: Pie is Delicious but not Nutritious <http://statisticalanalysisconsulting.com/graphics-for-univariate-data-pie-is-delicious-but-not-nutritious/>

*Thank you. This figure is actually SUPPLEMENTARY Figure 3, and has now been changed to a horizontal bar graph.*

Peter Flom

-----------------------------------------------------------

Reviewer #2: The paper is an important contribution to the field of disease-related economic impacts and priority setting. It reads well.

Below are a number of specific comments.

1. Title/Abstract: New Zealand should be mentioned in both the title and the abstract.

*Author response: Thank you. Title now changed to:*

*Disease-related income and economic productivity loss: longitudinal analysis of individual-level data for ~~an entire country~~ all New Zealanders*

*First line of “Methods and Findings” changed to:*

*We used linked health-tax data for all New Zealanders from 2006-07 to 2015-16 for 25-64 year-olds (22.5 million person-years).*

2. Introduction:

More general background information should be given on New Zealand, including: summary economic indicators (e.g. GDP per capita, income distribution, level of inequality); population numbers (e.g. total, proportion of old vs. young, distribution by ethnic group); health indicators (e.g. life expectancy at birth, composition of burden of disease); and health system characteristics (e.g. level of public vs. private financing, type of health system, what kind of insurance schemes?).

*Author response. We have added the following paragraph to a new first section “Population” in the Methods:*

*“New Zealand has a population of 5 million people with a median age of 38, 16% of the population aged 65 years and older, and a life expectancy of 81.4 years. Most New Zealanders are of European extraction but with sizeable numbers of Māori (Indigenous population; 16.5%), Asian (15.1%) and Pacific peoples (8.1%; 2018 Census data). The GDP per capita in 2018 was about US$42,000, ranked about 30th among all countries. Over 80% of total health expenditure is Government funded (through tax revenue). Regarding income protection, a separate accident insurance corporation exists that will cover 80% of one’s wage or salary whilst incapacitated, but income protection in the event of sickness is patchy comprising: accrued sick leave from one’s employer; income protection insurance from private insurance companies; and a relatively low publicly funded sickness benefit safety net.”*

3. Methods:

* Datasets: The authors should provide more details (and possibly references) on how the linkage between health and income data was conducted.

*Author Response: This paper at IJE [10] describes the core spine in detail. It was cited in Methods, but to draw the readers eye more to it, we have emphasized it:*

*“The health and income data are available to users of the Statistics New Zealand (SNZ) Integrated Data Infrastructure and are pre-linked using a resident population spine (data profile and methods published elsewhere [10]).*

* Analyses: This part is critical to the understanding of the methods and is currently too succinct and vague. More details should be provided with respect to the two steps behind the analysis:

(i) regression modeling and (ii) cause-deleted analyses. For instance: for (i) model equations should be given and described; and for (ii) the estimation of 95% uncertainty ranges based on (i) (I presume based on the regression coefficients?) should be explained.

*Author response. Thank you. We have:*

(i) *Removed the last paragraph on covariates under the “fixed effects regression modelling” sub-heading, and weaved it into this fuller text including the general regression model:*

*“The fixed effect regression model is:*

$$y_{it}=\beta_{1}x_{it}+a_{i}+u_{it}, t=1,2,3,\ldots\ldots\ldots..T$$

*Where:*

$y_{it}$ *is the dependent variable, income of the individual i (inflation adjusted) in time period or tax year t;*

$x_{it}$ *represents:*

- *the independent variables (i.e. main effects for time varying covariates [disease-phase (nil, first year, prevalent and last year of life if dying of the disease); tax year (categorical); five-year age-group (categorical); deprivation quintile (categorical); and a dummy variable for dying in that year if dying of a disease not included in model;*
- *interactions of the disease-phase variable (our ‘main exposure’ or variable of interest) with: a four-level age category covariate; ethnicity (Māori, Pacific, Asian and European Other; time invariant characteristics can be included in interactions with time varying covariates, here because of likely varying income loss by ethnicity); and deprivation.*

$\beta_{1}$ *represents the coefficient for the independent variables (with the standard error used to generate 95% CI); and*

$a_{i}$ *is the unknown intercept for each individual;*

$u_{it}$ *is the idiosyncratic error term.”*

(ii) *Yes, the 95% confidence intervals are based on regression coefficient standard errors. See above underlined text.*

4. Results:

* Could the authors report also per capita numbers for the estimates of disease-related costs?

*Author response. The results summarized in the second paragraph of the Results, and in Figure 1, are per capita costs. The above fuller description of the FE regression model will hopefully make this clearer. We have also:*

- *Altered the second sentence of second para of Results to read: “These per capita income losses represent the reference case person…..”*
- *Altered the Figure 1 caption heading to read: “Average per capita income loss (US$ 2018) per year….”*

* It would be interesting to see how the results (per disease) are correlated (or not) to the burden of disease by cause (e.g. using disease prevalence estimates).

*Author response: Yes, this is interesting. Below is a scatterplot plot for income gain by deleting diseases, with both YLDs and DALYs (now included as Supplementary Figure 4). Both the x-axis and y-axis are on the log scale. There is a clear correlation between the cause-deleted income loss and YLDs and DALYs (below; using DALYs and YLDs in 2011 in NZ as estimated by the GBD, for 25-64 year olds; source* [*www.GHDx.org*](http://www.GHDx.org)*). This is in large part because both the cause-deleted income loss and the YLDs/DALYs are driven by prevalence (i.e. a structural correlation). However, it is worth a mention in the Discussion, so we have added this sentence:*

*“Unsurprisingly, there is a strong correlation between aggregate disease-related income loss and health loss from the same conditions measured in disability adjusted life years and years of life lived with disability – due to both being largely driven by the prevalence of the condition (Supplementary Figure 4).”*

Cause-deleted income gain, plotted against YLDs and DALYs in 2011 from the GBD for 25-64 yr olds in NZ

DALYs or YLDs

| Income gain in US$ millions from deleting disease | Income loss - DALYs | Income loss - YLDs |
| --- | --- | --- |
| 115.6 | 86223.1 | 5550.05 |
| 12.8 | 10084.9 | 522.626 |
| 7.4 | 15123.9 | 183.781 |
| 35 | 12777.5 | 1280.52 |
| 9.4 | 2164.41 | 751.8 |
| 403.6 | 48134 | 6040.83 |
| 89 | 25832.5 | 1001.43 |
| 73.8 | 9674.43 | 2479.72 |
| 76.1 | 18709.6 | 11410.6 |
| 23.7 | 10454.8 | 5338.54 |
| 56.4 | 6473.59 | 5521.6 |
| 190.8 | 14048.8 | 8126.59 |
| 26.4 | 4143.74 | 375.037 |
| 39.5 | 6005.37 | 5651.5 |
| 34.1 | 6833.67 | 6460.54 |
| 16.9 | 5203.26 | 1538.03 |
| 167.1 | 10150.2 | 7150.6 |
| 168.9 | 30899.2 | 25472.7 |
| -55.6 | 19721.6 | 19721.6 |
| 352.6 | 83246.2 | 82208.1 |
| 39.3 | 4377.6 | 4377.6 |
| 6.7 | 1416.39 | 1212.94 |
| 773.7 | 60829.5 | 60818.7 |
| 336.9 | 36446.3 | 36446.3 |
| 147.7 | 6176.39 | 4855.4 |
| 80.6 | 7845.35 | 7845.35 |
| 89.7 | 95035.9 | 52216.8 |
| 49.7 | 12124.4 | 12124.4 |

* Could the authors pursue an analysis by socioeconomic status? That would be useful, in particular, given that, besides variations in disease prevalence, people may have different insurance coverage and schemes varying with income and socioeconomic status.

*Author Response: We agree. And this is being pursued as a separate additional publication (using the Kakwani index). We have added the following sentences to the end of the paragraph in the Discussion that touches on inequalities:*

*“Our results demonstrate inequalities in income loss by gender. Absolute income losses by disease are generally higher for males than females, but relative income losses are similar – reflecting known inequities in pay between males and females. If results such as ours are to be used in prioritizing health interventions based on their ability to improve individual incomes and aggregate productivity, we recommend careful attention to and correction for structural societal inequalities. A similar argument applies to diseases with varying rates by ethnicity and socioeconomic position; income loss by disease will vary in at least one of absolute or relative terms by socioeconomic position. Also, there may be sub- or super-additive impacts on income loss from having two, three or more conditions compared to the independent and separate (unconfounded) income loss for each condition as reported in this paper. These questions were beyond the scope of this paper, but will be pursued as additional follow-up publications.”*

5. Discussion:

* It would be good to have more explanations for the findings that are highlighted in the paper. The comparison with Norway is interesting but insufficient.

The authors should balance the epidemiological/burden of disease factors that may explain their results, with also some more fundamental health system issues (e.g. coverage of insurance and copayment, which may vary by disease condition). In other words, I believe the findings are highly contextual and depend on the local health system and levels of social protection: what is covered (e.g. specific disease treatments) by specific insurance programs and sick leaves? This should be better acknowledged in the context of what is known of copayments, sick leave policies, per disease/condition and income group/profession in New Zealand.

*Author response: We have added the following paragraph to the Discussion:*

*“Regarding generalizability to other high-income countries, the duration and generosity of employer sick leave may vary with other countries; in our analysis employer funded sick leave was not able to be ‘seen’ as it is simply part of salary or income. Therefore, the reader will need to be aware of this if their conceptualization of income loss (or productivity loss) includes employer sick pay. Second, the extent of assistance provided to people with health conditions to return to (paid) work varies by country. Third, the extent of disease-related income loss is likely to vary with the unemployment rate. If unemployment is high, then disease-related income loss is likely to be higher due to a greater pool of competitors for the same job (but conversely (and rather brutally) the productivity loss to society will be less as the sick person is more easily ‘replaced’ in the workforce). Nevertheless, the general patterns we observe likely hold in other countries – and our study is a valuable template for future comparison studies..”*

* Based on the previous point, it would be good to reflect on how the same analysis would likely differ in other countries with very different health systems than New Zealand.

*Author response: Please see above.*

* A better explanation could be provided with respect to why this approach can be used for priority setting: Is this something that can be ethically justified or not? And how such analyses could complement (or not) burden of disease arguments for example?

*Author response: We have added this text at the end of the Discussion:*

*“We argue that such comparability of estimates by disease, especially by phase of disease, at least within one country, opens a useful policy door to estimating the impact of interventions (e.g. salt reduction in bread that lowers stroke and ischaemic heart disease rates, e.g. obesity reduction programmes; e.g. treatments) on income loss in addition to the usual health gain and health expenditure impacts. Such additional analyses should be a useful adjunct to prioritize health interventions, if a desirable additional impact of interventions in the health sector is improvements in economic productivity. However, such analyses must be handled carefully, given the potential equity implications (e.g. gender as above).”*

*Please see above regarding burden of disease comparisons.*

-----------------------------------------------------------

Reviewer #3: Dear Authors,

Thanks for the opportunity to review your manuscript entitled, Disease-related income and productivity loss: longitudinal analysis of individual-level data for an entire country". Estimating income loss directly attributed to chronic disease is an important research area, especially from a population-level perspective. A highlight of the paper was the availability of linked health and income data which the research team uses to estimate income loss. Overall, the paper was interesting. However, there are several areas of improvement which I suggest in the following comments.

Background

*	The authors note that their study is novel because it is one of the first to estimate the impact of comorbidity on income loss. It is important to acknowledge the large body of research which has estimated the impact of specific chronic diseases on income and employment. Additionally, there are also studies that have examined the relationship between comorbidity and employment at the population level (e.g., US National Comorbidity Survey, US National Health Interview Survey, Canadian longitudinal National Populational Health Survey). It would have been helpful for the authors to dig deeper into the existing research and describe the specific gaps their research is filling. Also, through a more comprehensive synthesis of existing research, the authors could highlight the uniqueness of their research approach and methodology.

*Author response: The Introduction already addresses many of these points, e.g.:*

*“Studies often focus on a single disease, limiting the ability to compare across different diseases. In addition, confounding by comorbidities (or other diseases) is often ignored in studies that estimate income loss from one disease in isolation (e.g. ischaemic heart disease [11], rheumatoid arthritis [12], cancer [3]) leading to likely over-estimation of income loss for a given disease. Put another way, if such studies were undertaken separately for all diseases the sum of income loss across these studies would (likely greatly) exceed the actual income loss from all diseases considered together.*

*On the other-hand, some studies only include income loss or productivity loss from deaths [13, 14] tallying up all income loss had the person hypothetically lived to (say) 65 years of age (which also ignores competing morbidity and competing mortality risk).*

*None of the previous studies, with the possible exception of Kinge et al (2017), include multiple diseases. The uniqueness of our study is outlined in the Introduction (and further emphasized with the underlined addition):*

*“To overcome the limitations in previous studies on disease-related income loss, and provide income loss estimates that are comparable across diseases, we used population-wide panel data on disease and injury events linked to income data– for all diseases simultaneously to overcome confounding, which we believe is unique. We estimated income loss whilst the subject is alive or in the tax year of their death, for adults aged 25-64 years, adjusting for comorbidity. There is also likely to be further confounding by socioeconomic position (SEP), in that SEP causes both variations in disease rates and income (loss); we aimed to estimate the income loss a person would have avoided had they counterfactually not developed the given disease. Our inference target was the average citizen or total population – not just those employed; accordingly, we estimated income loss estimates averaged across all citizens. Specifically, our research objectives were to determine: 1) individual-level disease-specific income loss estimates by phase of diagnosis; and 2) population-level estimates of total income loss by disease, and the ranking of disease contributions to income loss in the total population.”*

*We do, however, agree that emphasizing that even Scandinavian studies are mostly single disease (despite population registries) is a useful addition to the Introduction:*

*“Even studies from Scandinavia, with population-wide disease registers linked to taxation or employment data, have also focused on single diseases such as diabetes [15], breast cancer [16], and injury.[17]”*

*	In the background it would also be important for the authors to provide support for their study objective. Why is phase of diagnosis important when it comes to estimating income loss?

*Author response: Phase is useful in prioritization, as one can position the income loss by time post-diagnosis, which is important for the stream of income impacts over time. We do not think this paper is the place to expound this in detail, but in response to Referee #2 we have added a paragraph at the end of the Discussion on prioritization – and added in a mention of ‘by phase estimates’ there.*

I was expecting the authors to include specific study objectives or hypotheses comparing different health conditions.

*Author response: This “comparing different health conditions” was implicit in our existing objectives. However, to make this more explicit, we have revised the second objective as stated in the last sentence of the Introduction as underlined:*

*“….2) population-level estimates of total income loss by disease, and the ranking of disease contributions to income loss in the total population.”*

I was also expecting objectives or hypotheses related to having a single chronic disease versus one or more chronic diseases.

*Author response: We agree this is important – and it is the focus of a separate follow-up paper, and is addressed with this sentence in the Discussion:*

*“Also, there may be sub- or super-additive impacts on income loss from having two, three or more conditions compared to the independent and separate (unconfounded) income loss for each condition as reported in this paper.”*

Methods

*	The analytical process that the authors take is unique. However, the authors assume that the reader will completely understand the statistical methods and approaches that they used. For greater clarity, I would suggest that the authors consider adding some more information to this section. I note several suggestions in the sections below.

*Author response: We agree that this work is innovative in the data and methods applied, but the analytical approach is not ‘unique’ – a net or excess costing approach is a recognized and standard approach as per references 17 to 20 in the paper.*

*As above, we have provided more detail on the FE regression method.*

*	First, the author draws on multiple registries and health-related datasets. Given that these appear to be different sources that authors should elaborate on how these datasets were harmonized in order to estimate the number of people with a chronic disease. Additional information would also be helpful to provide details on how the different registries/data sources were used to estimate comorbidity.

*Author response: Regarding harmonization, the data we used is in the Statistics NZ Integrated Data infrastructure, and as in response to Referee 1 above we have emphasized the data profile describing this resource. Regarding comorbidity:*

- *the ‘Diseases’ section in the Methods overviews disease classification, and Supplementary Table 1 provides (exhaustive) detail on how diseases were assigned*
- *we have added this parenthetic sentence at the end of the ‘Covariates’ section in the Methods:*

*“(We did not use a comorbidity index per se, but rather as all models were adjusted for all other diseases (be that the 14 or 40 disease level) we consider this adjustment for confounding by other diseases as equivalent to adjusting for comorbidity.)”*

*	The authors describe including 14 aggregate disease. How did they decide on which diseases they would include and which they might exclude? Did the authors chose to focus on commonly reported chronic diseases?

*Author response: We adhered to the disease and condition groupings used in the NZ Burden of Disease Study. This may not have been clear in our methods, so we have reworded the first sentence of the ‘Diseases’ section of ‘Variables’:*

*“We used the NZ Burden of Disease Study (NZBDS) condition groupings [18, 19] to select 14 aggregated and 40 disaggregate-level diseases or conditions (see Supplementary Table 1 for coding, Table 2 for categories NZBDS categories with insufficient data or no variation over time (dental and congenital) were excluded).”*

*	In their analytical models, were the authors able to control for disease management? Certain chronic diseases can have a significant impact on work if they are not treated with pharmacologic or non-pharmacologic approaches. These same diseases could have no impact on income generation if appropriate management is accessed.

*Author response: No. This was beyond the scope of what we proposed, and likely beyond the scope of the data. That said, we are also concerned about this for migraine, as per this text that was already included in the Discussion (with slight addition underlined):*

*“Migraine was the only disease that resulted in a (modest) increase in income, for females only, but with 95% confidence intervals excluding the null. Whilst it could be a chance finding, it is also not implausible: a diagnosis of migraine may lead to better treatment and more productivity; conversely, increased stress in the workplace due to longer hours or more responsibilities with career advancement (and therefore income) may trigger migraines.”*

*	Also, the authors examine the time varying nature of chronic disease. Were the authors able to capture the episodic nature of chronic disease? Certain conditions like depression, arthritis, multiple sclerosis can fluctuate on a weekly, monthly or yearly bases. Indeed, a period of disease flare will have a more significant impact on income generation. Could the authors account for this level of variability?

*Author response: For most conditions and diseases, other than injury and cancers (from which one was considered cured if surviving longer than the usual cure time), once diagnosed the person was considered to have that condition for the rest of their life.*

*We believe our inclusion of three phases of disease (diagnosis year, prevalent, and last year of life if dying of that disease) is actually a major step forward in this field. Additionally, including disease severity or ‘activity’ is a worthy extension, but was beyond the scope of this study.*

*	How did the authors account for comorbidity? If a person was living with more than one chronic condition, was there a method to determine which chronic condition had the biggest impact on income loss?

*Author response: In this paper, we focused ‘just’ on the separate and independent impacts of 14 or 40 diseases. As detailed above, we are examining dual or more diagnoses in a follow-up paper.*

*	I was curious to learn more about the ethnic group covariate. I found it troubling that members of the research team prioritized an ethnic group, when a participant noted being part of more than one ethnic group. Perhaps I misunderstood. Maybe the authors could clarify.

*Author response: This is an important issue in NZ.*

1) *If looking at ethnicity per se as the ‘exposure’ of interest, it is important to present results for ‘total’ ethnic groups (e.g. anyone self-identifying as Maori, anyone self-identifying as Pacific, etc.). In so doing, the groups are not mutually exclusive as people can have multiple ethnic groups.*
2) *But if ethnicity is a covariate, it may be adequate to use a prioritized variable (e.g. Maori, Pacific, Asian and European/other in that priority order). It will unlikely make much difference to the exposure-outcome association of interest (in this paper disease-income), and is vastly more tractable in regression analyses as the categorization is mutually exclusive.*

*The first author of this paper, Blakely, has 50+ peer reviewed papers addressing ethnicity through either 1) or 2) above. In this current paper, 2) is the best avenue – and acceptable practice.*

*In the interest of space, we do not think it is necessary in this paper to re-rehearse these points, but can do so if the Editor wishes us to.*

*	Similarly, more details on the material deprivation covariate would have been helpful.

*Author response: We have added this sentence after its first mention in the Methods:*

*“The NZDep measure is a principal components calculated index using nine census variables at a small-area (meshbock) level of about 100 people; proportion of income as benefit receipt; household income; housing tenure; sole parent family; unemployment; qualifications; household crowding; telephone access; and car access.”*

*	Can the authors provide a few more details on the net cost approach?

*Author response: We have added two sentences to the Methods:*

*“Conceptually, this excess costing approach examines how individual’s incomes vary year by year, corresponding to their disease status in each year. If the average difference in income in people’s first year of diagnosis of stroke (compared to their own pre-stroke years) was $5000 (adjusted for other changes over time such as other diseases and changing deprivation), then this is estimated income loss.”*

*	Also, the authors include interaction effects into the models. Perhaps they can elaborate on the interaction effects they included and how they align with study objectives they describe in the background sections.

*Author response: Interactions were not a research question per se in the paper, but important nevertheless. We have added these two sentences in the description of the FE model:*

- *“(These interactions were included to allow for likely variations in income loss by disease with age and ethnicity; whilst not a research question per se in this study, allowing for such interactions is important in the cause-deleted analyses below that estimate total income loss from disease across the population.)”*

*	I found the cause-deleted analyses to be an interesting approach. Did they authors develop this approach themselves? Is it commonly used in economic estimates of a similar nature.

*Author response: A cause-deleted analysis is sometimes used in epidemiology.*

Results

*	I found the results section a bit hard to follow. I would suggest making the findings clearly align with the study objectives.

*Author response: We have added two sub-headings to orientate the reader:*

*Objective 1: individual-level disease-specific income loss estimates by phase of diagnosis*

*Objective 2: population-level estimates of total income loss by disease*

*The Results text already followed this structure – it just needed the sub-headings.*

Discussion section

*	A comprehensive discussion of the implications of the study findings was missing. I was curious to know the author's interpretation of the findings. Why was income loss greater for males? For example, why would dementia be attributed to the greatest income loss post diagnosis? Is it a clinical impact? Age-related impact? Or perhaps dementia is more likely to affect people earning a greater amount of income to begin with? Also, I am also curious to know why migraines may have been associated with an income gain. I would also suggest the authors provide more insight into the implications of the findings.

*Author response: We have expanded the Discussion to address various Reviewer comments above; e.g. added detail on gender differences in income loss and around study limitations/generalizability. The issue of the atypical migraine results are also discussed. We do think the curious finding for dementia needs comment, hence we have added the following sentence to the first paragraph of the Discussion:*

*“The finding of high per capita income loss for people with dementia in their first year of life and if prevalent was unexpected, but also based on relatively few people (Supplementary Table 2); it may be that dementias before the age of 65 selectively impact the ability to work of high income occupations.”*

*Beyond this, our preference with the Discussion Section is to try to be concise and comment only on the key findings as opposed to a full disease-by-disease commentary. Nevertheless, if the Editor desires an even more detailed Discussion – we would revise accordingly.*

The finding of high per capita income loss for people with dementia in their first year of life and if prevalent was unexpected, but also based on relatively few people (Supplementary Table 2); it may be that dementias before the age of 65 selectively impact the ability to work of high income occupations.

-----------------------------------------------------------

Any attachments provided with reviews can be seen via the following link:

__________________________________________________

In compliance with data protection regulations, you may request that we remove your personal registration details at any time. (Use the following URL: <https://www.editorialmanager.com/pmedicine/login.asp?a=r>). Please contact the publication office if you have any questions.

**References**

1	Gunasekara FI, Richardson K, Carter K*, et al.* Fixed effects analysis of repeated measures data. *Int J Epidemiol* 2014;**43**:264-9.

2	Jacobson LS, LaLonde RJ, Sullivan DG. Earnings losses of displaced workers. . *The American Economic Review* 1993;**83**:685-709.

3	Zajacova A, Dowd JB, Schoeni RF*, et al.* Employment and income losses among cancer survivors: Estimates from a national longitudinal survey of American families. *Cancer* 2015;**121**:4425-32.

4	Barlow WEP. Overview of Methods to Estimate the Medical Costs of Cancer. *Medical Care July* 2009;**47**:S33-S6.

5	Fireman BH, Quesenberry CP, Somkin CP*, et al.* Cost of care for cancer in a health maintenance organization. *Health Care Financ Rev* 1997;**18**:51-76.

6	Lipscomb J, Barnett PG, Brown ML*, et al.* Advancing the science of health care costing. *Med Care* 2009;**47**:S120-6.

7	Mariotto AB, Yabroff KR, Shao Y*, et al.* Projections of the cost of cancer care in the United States: 2010-2020.[Erratum appears in J Natl Cancer Inst. 2011 Apr 20;103(8):699]. *Journal of the National Cancer Institute* 2011;**103**:117-28.

8	Blakely T, Kvizhinadze G, Atkinson J*, et al.* Health system costs for individual and comorbid noncommunicable diseases: An analysis of publicly funded health events from New Zealand. *PLoS Med* 2019;**16**:e1002716.

9	Rothman K, Greenland S, Lash T. *Modern Epidemiology*. Philadelphia: Lippincott Williams & Wilkins 2008.

10	Milne BJ, Atkinson J, Blakely T*, et al.* Data Resource Profile: The New Zealand Integrated Data Infrastructure (IDI). *Int J Epidemiol* 2019.

11	Herrin J, Cangialose CB, Boccuzzi SJ*, et al.* Household Income Losses Associated With Ischaemic Heart Disease For US Employees. *PharmacoEconomics* 2000;**17**:304-14.

12	Wolfe F, Michaud K, Choi HK*, et al.* Household income and earnings losses among 6,396 persons with rheumatoid arthritis. *The Journal of Rheumatology* 2005;**32**:1875-83.

13	Carter HE, Schofield D, Shrestha R. The long-term productivity impacts of all cause premature mortality in Australia. *Australian and New Zealand Journal of Public Health* 2017;**41**:137-43.

14	Zheng H, Ehrlich F, Amin J. Productivity loss resulting from coronary heart disease in Australia. *Applied Health Economics and Health Policy* 2010;**8**:179-89.

15	Persson S, Johansen P, Andersson E*, et al.* Days absent from work as a result of complications associated with type 2 diabetes: Evidence from 20 years of linked national registry data in Sweden. *Diabetes Obes Metab* 2020;**22**:1586-97.

16	Dahl FA, Šaltytė Benth J, Aas E*, et al.* Economic productivity loss due to breast cancer in Norway – a case control study using the human capital approach. *Nordic Journal of Health Economics* 2017;**5**:70-83.

17	Haikonen K, Lillsunde PM. Burden of Fire Injuries in Finland: Lost Productivity and Benefits. *J Public Health Res* 2016;**5**:705-.

18	Ministry of Health. Health Loss in New Zealand: A report from the New Zealand Burden of Diseases, Injuries and Risk Factors Study, 2006–2016. Wellington: Ministry of Health 2013.

19	Ministry of Health. Health Loss in New Zealand 1990–2013: A report from the New Zealand Burden of Diseases, Injuries and Risk Factors Study. Wellington: Ministry of Health 2016.
