# Supplementary material for: Disease-related income and economic productivity loss in New Zealand: A longitudinal analysis of linked individual-level data
Source: PLoS Med. 2021 Nov 30;18(11):e1003848. doi: 10.1371/journal.pmed.1003848 (PMC8631646; doi:10.1371/journal.pmed.1003848)
Supplement: Attachment — Submitted filename: Plos Med Income Loss 2nd Rev_Response to Reviewers.docx [file pmed.1003848.s014.docx]

Dear Editor

22 Jan 2021

**Re:	Disease-related income and economic productivity loss: longitudinal analysis of individual-level data for an entire country (PMEDICINE-D-20-03416R1)**

REQUESTS FROM EDITORS:

1. Please respond to and, where possible, address the reviewer’s final comments. Additional comments from the Academic Editor: “I would emphasize that the authors should clearly explain the pros and cons of converting the continuous predictors into categorical variables. Despite their reasonable response to reviewer #1 on this issue, the authors do need to convince the more statistically oriented reader why it is that categorical variables are preferred in this situation. Ideally, they should show that a sensitivity analysis with continuous variables produces qualitatively similar results.”

*Author response: please see our detailed response to reviewer #1. We propose that reviewer comments, and or replies, are made accessible to readers (as offered by Plos Medicine routinely) as the mechanism to “… convince the more statistically oriented reader”.*

2.	Financial Disclosure: Please add “The funders had no role in study design, data collection and analysis, decision to publish, or preparation of the manuscript.”; or explain otherwise.

*Author response: Done – added to Acknowledgements.*

2. Data Availability Statement: Correct to “…data…are available…”

*Author response: Done*

3. Title: Please amend to: “Disease-related income and economic productivity loss in New Zealand: A longitudinal analysis of linked individual-level data”

*Author response: Done*

4. Short title: Please amend to: “Disease-related income loss in New Zealand”

*Author response: Done*

5. Please add line numbers in the margin throughout.

*Author response: Done*

7.	Abstract:

a.	Reword to “…adjustment for confounding are lacking, to our knowledge.”

b.	Please briefly quantify these results: “Mental illness also had high income

c.	losses in the year of diagnosis. Similar patterns were evident for prevalent years of diagnosis.”

d.	Please make more explicit that all $ values shown are USD.

e.	At the end of the ‘Methods and findings’ subsection, please reword the limitations to be more explicit: “The limitations of this study were…” (or similar). You can also be more concise in describing the limitations by removing the examples after each “e.g.”. However, you can also add in other limitations such as omitting sick pay or migrant workers.

f.	Conclusions: Please begin with “In this longitudinal study, we found that…”

*Author response: Done. Regarding more detail in abstract for mental illness, we added: “diagnosis (average of about $4000 per year for males and $3000 per year for females, for four sub-categories of depression and anxiety; alcohol related; schizophrenia; and other)”.*

8.	Page 3: Please rename to simply “Author Summary”.

*Author response: Done*

9.	Author Summary: Please clarify that $ is USD.

*Author response: Done*

10.	Please use the "Vancouver" style for reference formatting, and see our website for other reference guidelines <https://journals.plos.org/plosmedicine/s/submission-guidelines#loc-references>. Citations should be in square brackets, before punctuation, and not superscript, eg. “…[1,2].”

*Author response: Done*

11.	The terms gender and sex are not interchangeable (as discussed in <http://www.who.int/gender/whatisgender/en/> ); please use the appropriate term.

*Author response: Done*

12.	Methods:

a.	Please provide a copy of your analytical plan as Supporting Information, and cite the file under ‘Analyses’.

*Author response: We addressed this in the last set of comments. By including this text under ‘Analyses’ in the Methods section:*

*“Our analytical plan for this study was to follow the analyses we previously conducted (and published in this Journal) for disease-related health system expenditure.[1] We however deviated from this previous approach to use fixed effects (FE) regression analyses for the ‘main analyses’, reporting ordinary least squares (OLS) regression in sensitivity analyses due to concerns about residual confounding (below).”*

b.	Please mention that separate ethical approval was not required for your study.

*Author response: The following is added to beginning of the Methods:*

*“The study was approved by Statistics New Zealand for undertaking in the SNZ Integrated Data Infrastructure, and separately approved by the University of Otago Ethics Committee.”*

c.	Please cite your RECORD checklist: "This study is reported as per the RECORD guideline (S1 Checklist)."

*Author response: Done*

13.	Page 10: Please make the Results subheading more distinct.

*Author response: Done*

14.	Discussion:

a.	Please fix broken citation “(Error! Reference source not found.)”

b.	Please rephrase to avoid nesting brackets: “…(but conversely, and rather brutally, the productivity loss to society will be less as the sick person is more easily ‘replaced’ in the workforce).”

c.	Please add a one-paragraph conclusion to the end, summarising your study.

*Author response: Done. New last paragraph reads:*

*“In summary, we used a unique data base of repeated disease and tax income measures on an entire population. Fixed effects regression estimated the within-individual change in income when developing disease among 25 to 64 year olds. Income loss among individuals developing disease was highest for dementia (noting this was dementia onset before the age of 65), followed by mental illness. From a total population perspective, combining the prevalence of disease with the income loss estimates per individual, the three largest causes of income loss were mental illness, cardiovascular disease and musculoskeletal diseases. Our study is a major advance, including all diseases simultaneously and quantifying within-individual income loss. We encourage other countries to also conduct such comparable analyses, and then further to trial including such income loss estimates as additional considerations in intervention prioritization with policymakers.”*

15.	Please remove funding information from the Acknowledgements.

*Author response: Done*

16.	References: Please remove bold and italic formatting. First 6 authors should be shown, followed by “et al.”

*Author response: Done*

17.	Please complete all sections of the RECORD checklist; state ‘n/a’ for items not applicable (though this should be rare).

*Author response: Done*

18.	Please provide more access details (eg. volume/issue/pages, DOI or URL) for references 15, 16, 17, 18.

*Author response: Done using ISBN numbers for 16-18; URLs can be provided, but quite long.*

COMMENTS FROM REVIEWERS:

Reviewer #1: NOTE: Much of what I have below is a matter of informed opinion rather than absolute fact. I think my opinions are justified, but other statisticians might disagree. The authors clearly know a lot about statistics. But the editors asked for my opinions again, rather than those of some other statistician, so, here they are.

The authors have responded to my comments, but there are some remaining issues:

I wrote

Another general concern is what population you are inferrring to. You have the whole population of New Zealand. Are you inferring to the rest of the world? That seems likely to be very wrong. Or perhaps to other high-income countries? Or what?

Some statisticians justify this by inferrring to some "superpopulation" -- I am not a big fan of this, but it's not outright wrong. But you need to specify what the population or super population is.

Author response:

We are inferring to the total NZ population, then talking about generalizability in the Discussion (see below).

My response: You cannot infer to the sample that you have. If you try, then all p values would be exactly 0, all CIs would be point estimates. The p values and CIs have to refer to some larger group. Generalizability is a different issue - that's one of how well analysis done in New Zealand would apply to other places. So, the authors could do a purely descriptive study of NZ -- I think that would be fine.

*Author response: We respectfully disagree. We have encountered this issue many times before, as we often utilize national data-bases. Some would argue (and we agree an argument can be made) that we have no sample – therefore no p values or CI are required. However, if you think about it, there are underlying constructs of true rates of disease, and such like. Each year, through a Poisson resembling process, events occur. These events, the historical record, are just one realization of how the world might have unfolded. We can counterfactually imagine last year playing out in different ways.*

*From a more pragmatic point of view, for uncommon diseases, or strata of disease by gender by age by income .. etc… one inevitable gets to small numbers of events. Even if they are all the events in a population in a given year, they are still prone to random or chance variation year to year.*

*Finally, the above arguments have been rehearsed elsewhere. The common place I point to is Rothman et al[2] top of page 149:*

*“Nevertheless, it is standard practice to treat all epidemiologic studies, including cohort studies, as having sampling error. [Participants] … are viewed as a figurative sample… even if all individuals in a population are included….” Followed by series of points similar to above.*

*If the editor wishes, we can state our approach in the Methods – but we think it not necessary, and a distraction from the main fare.*

-------------------------

I objected to categorizing continuous variables. The authors responded, first, with regard to age:

We tried extensive alternative specification of variables in analyses prior to submission. Continuous variants for age (say) were problematic due to non-linearities by age, requiring separate specification by each disease in interaction terms and such like. We had problems with (say) quadratics for age generating predictions that were far from the observed data at the younger and older age groups. For model parsimony reasons we used categorical variables. We note that with the entire population of NZ included, that study power is good (i.e. we were not sacrificing many degrees of freedom). Second, we note that with covariate adjustment in epidemiology and biostatistics, it is common practice to use categorical variables - with a common rule of thumb being that more than 4 or 5 categories captures the majority of confounding.[9] Third, we note that with cause deleted analyses categorical variables are perhaps a bit easier conceptually.

My response:

The authors point about this being a common method is entirely correct. But I'm not sure that's an argument that it is a good method.

I agree that quadratics are problematic; I would suggest a spline. It's true that splines can be a bit hard to interpret (although graphs can help). The problem with categorizing the variable at arbitrary points is that there is no way to know if those are the right points, And, since the authors note that the data are nonlinear, using categories imposes linearity within each category. Regarding parsimony - well, you certainly have ample degrees of freedom to play with, but parsimony is, indeed, a good aim. But, from what I can tell, the parsimony gain is from assuming linearity between categories. I'm not sure that is worth it. Perhaps a compromise would be for the authors to do an analysis with splines and see if it is much different than the one with categories, then proceed accordingly.

More generally (and this is more for the PLOS editors than the authors), I think it is good for authors who know what they are doing (as these authors clearly do) to use advanced methods where appropriate. PLOS Medicine wants to advance medical techniques by publishing articles by authors who are experts in new methods. So, I think, they should also want to advance statistical methods.

For the other categorical variables I think the authors' response makes sense.

*Author response:*

*We respectfully do not think that the analyses need representing with splines by age; we propose that these referee reports and our responses are made accessible to readers (as routinely offered by Plos Medicine), and act as the record requested by the editor, namely that: “… the authors should clearly explain the pros and cons of converting the continuous predictors into categorical variables. Despite their reasonable response to reviewer #1 on this issue, the authors do need to convince the more statistically oriented reader why it is that categorical variables are preferred in this situation.” A detailed explanation follows:*

*First, the exposure in our study is disease phase, a nominal and categorical variable. Our outcome is income, which is already treated as continuous. Potential mis-specification of covariates, such as age, is less concerning than mis-specification of exposures and outcomes.*

*Second, we use fixed effects analysis. Thus, from the perspective of confounding, correct specification of time varying confounders is what matters most. Age does vary with time, but in a completely predictable manner – it increases by 1 with each annual wave. Our analyses include dummies by year/wave, and five-year age group. Therefore, the age covariate is just picking up the small impact of increasing age every five years, over and above annual changes. Thus, it seems unlikely that there will be gain in continuous specifications of age (or splines) as a main effect or potential confounder. Indeed, in sensitivity analyses where we used continuous age and age-squared (as we refined our method), if there was a notable impact on our exposure (disease) – outcome (income) association of interest, it was actually a model mis-specification with predicted values deviated notably from observed values at either end of the age spectrum.*

*Third, and importantly, age does modify the effect of the disease – income association. (i.e. the association of disease – income varies by age). As described in the Methods of the main paper, we specify this interaction using wider age groups (25-39, 40-49, 50-59 and 60-64-year-old categories) – using narrower age categories results in very small cell sizes, wide imprecision, and (we suspect) no reliable gain in accuracy.*

*To assist the referee and reader, below is a graph of the income difference for females with prevalent IHD compared to healthy females. The main effect for prevalent IHD is an income loss of US$2765, compared to healthy females. Given the interaction of age with disease phase, this US$2765 loss applies to 50-59 year olds (the age referent category). Below are the income losses for the three other age groups include in the interaction term with age.*

*Would the use of a continuous age variable (transformed, or splines) substantially improve our paper? “No” for analyses that do not present results by age, as the unders and overs by not stratifying more finely or allowing (say) some spline function by age will average out. Figure 1 and Table 2 are therefore fine. Figure 2 (cause deleted income loss by five-year age groups) might be improved. However, we do not give exact (i.e. $ values) cause-deleted income loss by age; the Figure 2 is merely to show the patterning. Table 2 gives $ values for cause-deleted income for all ages combined – so for actual $ estimates they are across all ages.*

*Fourth, and as in our initial response to referee #1, we did try continuous variants of income prior to submission (linear plus quadratic age, each as main effects [see comments above] and each interacted with disease phase). But it was unstable, and unreliable with quadratics (e.g. income loss at the age extremes was often quite removed from the observed income loss). Given often times sparse data (less common diseases, diagnosis year and especially year of death), you have to be seriously concerned about over-fitting compared to what we have done – a simple four level categorical variable of age interacted with disease phases. We concur splines would be statistically better, but given we have four age groups routinely fitted, and three age knots would seem a likely maximum, results would likely differ little.*

*Finally, the opportunity cost to repeating all these analyses with (say) age splines is non-trivial. Accessing data in the SNZ IDI is a major undertaking; it is not just on our own PCs. Fitting of age splines to 40 diseases, for both males and females, interacted with three disease phases is 240 model fittings.*

*For all these reasons, we do not think it warranted to include age splines (or other transformation of continuous age).*

------------------

Finally, we disagree about two of the graphics. I still think I am right, but this is (partly) a matter of style and I leave it to the editors to decide between the different graphs. Certainly the authors graphs are common choices.

*Author response: we prefer to keep with our graphs, thanks.*

Peter Flom

Reviewer #2: The authors have addressed my comments.

Reviewer #3: Dear Author,

Thank you for your revisions. You addressed all of my comments.

I would suggest that you carefully review grammar and formatting of the paper to improve flow and readability. Also, there were a few issues with the referencing that should be fixed.

*Author response: we have proof-read the paper in its entirety again – presumably any residual issues can be ironed out at copy-editing.*

1	Blakely T, Kvizhinadze G, Atkinson J*, et al.* Health system costs for individual and comorbid noncommunicable diseases: An analysis of publicly funded health events from New Zealand. *PLoS Med* 2019;**16**:e1002716.

2	Rothman K, Greenland S, Lash T. *Modern Epidemiology*. Philadelphia: Lippincott Williams & Wilkins 2008.
