# Supplementary material for: Disease-related income and economic productivity loss in New Zealand: A longitudinal analysis of linked individual-level data
Source: PLoS Med. 2021 Nov 30;18(11):e1003848. doi: 10.1371/journal.pmed.1003848 (PMC8631646; doi:10.1371/journal.pmed.1003848)
Supplement: Attachment — Submitted filename: Disease-related income loss, Revision 3 letter.docx [file pmed.1003848.s015.docx]

12 October 2021

Dear Richard Turner,

**Re:	Disease-related income and economic productivity loss in New Zealand: A longitudinal analysis of linked individual-level data" (PMEDICINE-D-20-03416R3)**

My apologies for the delay in getting this back to you. We have been hit hard by COVID and other priorities.

Also, in the process of finalizing this paper, we have updated all the 2018 USD estimates to 2020 USD estimates, given time that has elapsed. I have not shown these changes in track changes to reduce clutter.

*Editor requests*

Please make that "these data" in the data statement. We note that you specify that "some restrictions will apply" to data access, and we ask that you briefly state the nature of these restrictions, e.g., that access might be restricted to researchers with suitable ethics approval for their planned study.

*The data access statement has been expanded to:*

*“These data are available in the Statistics New Zealand Integrated Data Laboratory. Details on how to access this data can be found at https://www.stats.govt.nz/integrated-data/apply-to-use-microdata-for-research/. Or contact SNZ at: access2microdata@stats.govt.nz.*

*Applicants to use the data will need ethics approval and a data integration approval from SNZ. At this point in time, only researchers based in NZ can access the data through either data laboratories on site at SNZ, and satellite-laboratories in a number of NZ institutions (e.g. hosted by NZ universities).”*

At line 23, for example, please adapt the phrasing to: "... cardiovascular ... endocrine ... and neurological diseases, ... and cancer".

*Done.*

Please restructure the early part of the Discussion section. The first paragraph should summarize the study's findings, so we suggest introducing a paragraph break where the findings are compared to those of a previous study.

*Thank you. We have moved out the ‘Norway’ sentence to begin a second paragraph on international comparisons and generalizability.*

Please ensure that reference call-outs precede punctuation (e.g., "... injury [12].") and do not contain spaces, e.g., at line 91 ("... loss from deaths [13,14] ").

*Done (without track changes on to reduce clutter).*

Please substitute "sex" for "gender" where appropriate, e.g., at line 431; in the RECORD checklist.

*Done*

Please use the style "age X years" consistently, e.g., at line 435.

*Done – I think; please just correct anything that I may not have done quite correctly.*

Sincerely

Tony Blakely (and on behalf of co-authors)
